# Can Foraging Ecology Drive the Evolution of Body Size in a Diving Endotherm?

**DOI:** 10.1371/journal.pone.0056297

**Published:** 2013-02-07

**Authors:** Timothée R. Cook, Amélie Lescroël, Yves Cherel, Akiko Kato, Charles-André Bost

**Affiliations:** 1 Percy FitzPatrick Institute of African Ornithology, Centre of Excellence (Department of Science and Technology–National Research Foundation), University of Cape Town, Rondebosch, South Africa; 2 Centre d’Études Biologiques de Chizé, Centre National de la Recherche Scientifique (Unité Propre de Recherche 1934), Villiers-en-bois, France; 3 Centre d’Écologie Fonctionnelle et Évolutive, Centre National de la Recherche Scientifique (Unité Mixte de Recherche 5175), Montpellier, France; 4 Institut Pluridisciplinaire Hubert Curien, Université de Strasbourg, Strasbourg, France; 5 Centre National de la Recherche Scientifique (Unité Mixte de Recherche 7178), Strasbourg, France; 6 National Institute of Polar Research, Itabashi-ku, Tokyo, Japan; University of Roehampton, United Kingdom

## Abstract

Within a single animal species, different morphs can allow for differential exploitation of foraging niches between populations, while sexual size dimorphism can provide each sex with access to different resources. Despite being potentially important agents of evolution, resource polymorphisms, and the way they operate in wild populations, remain poorly understood. In this study, we examine how trophic factors can select for different body sizes between populations and sexes in a diving endotherm. Dive depth and duration are positively related to body size in diving birds and mammals, a relationship explained by a lower mass-specific metabolic rate and greater oxygen stores in larger individuals. Based on this allometry, we predict that selection for exploiting resources situated at different depths can drive the evolution of body size in species of diving endotherms at the population and sexual level. To test this prediction, we studied the foraging ecology of Blue-eyed Shags, a group of cormorants with male-biased sexual size dimorphism from across the Southern Ocean. We found that mean body mass and relative difference in body mass between sexes varied by up to 77% and 107% between neighbouring colonies, respectively. Birds from colonies with larger individuals dived deeper than birds from colonies with smaller individuals, when accounting for sex. In parallel, males dived further offshore and deeper than females and the sexual difference in dive depth reflected the level of sexual size dimorphism at each colony. We argue that body size in this group of birds is under intense selection for diving to depths of profitable benthic prey patches and that, locally, sexual niche divergence selection can exaggerate the sexual size dimorphism of Blue-eyed Shags initially set up by sexual selection. Our findings suggest that trophic resources can select for important geographic micro-variability in body size between populations and sexes.

## Introduction

Divergent selection for exploiting different trophic resources can lead to divergence between animal populations or even to speciation [1]. In particular, different sizes of bodily traits between populations may provide different capacity in utilizing food. A larger body provides greater reaching capability, and larger mouth parts enable the handling and ingestion of larger prey items for example. Resource polymorphism may therefore provide variable fitness depending on the type of environment (e.g. [2], [3]). Despite its obvious central role in the theory of adaptive radiation, resource polymorphism remains an underestimated agent of biodiversity, partly because many polymorphisms are so subtle they are difficult to detect [4].

A corollary of resource polymorphism is resource sexual size dimorphism. In theory, sexual size dimorphism (SSD) may provide each sex with access to different resources, which may increase the fitness of some individuals, thus selecting for more SSD [5]–[7]. Although models suggest that sexual niche divergence selection could be widespread [8], convincing examples are relatively rare (e.g. [9]–[11]). In particular, acceptance that SSD may evolve because of differing trophic adaptations in males and females is often hindered by the methodological difficulty of demonstrating that an observed dietary divergence is not simply a morphological consequence of SSD evolved under sexual selection [5], [12] or fecundity selection [5], [13]. The actual significance of sexual niche divergence selection as a cause of SSD is therefore still under debate [7], [8], [14]–[16].

**Figure 1:**
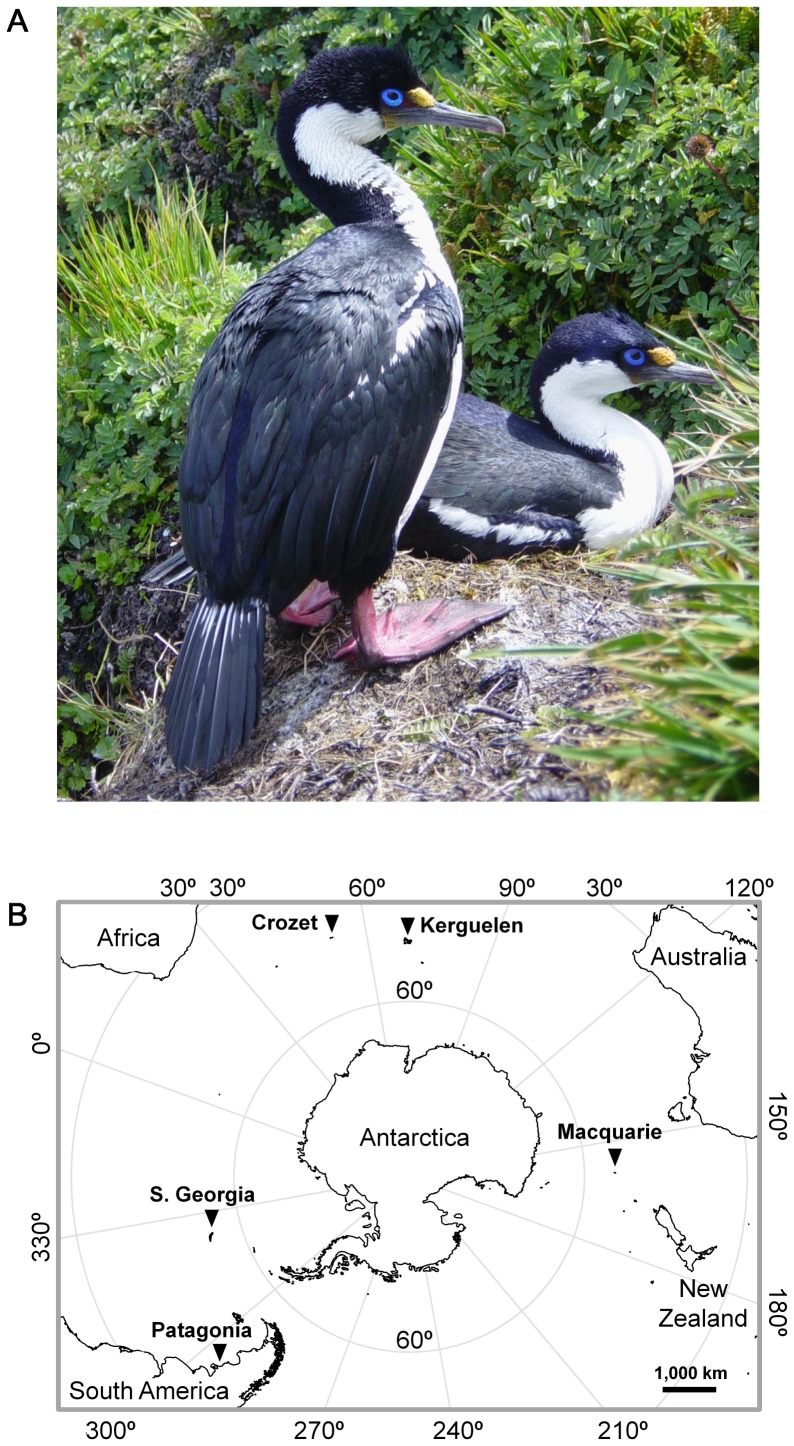
Distribution of Blue-eyed Shag colonies. (A) Blue-eyed Shag breeding pair at Crozet, with male standing in the foreground (photo: Carolyne Dodelinger). (B) Position of the Blue-eyed Shag studies that were reviewed in this paper.

Vertebrates are an interesting group for studying the evolution of body size in relation to resource because many species simultaneously display SSD and sex segregation in foraging behaviour [7], [17]–[19]. The Blue-eyed Shags are unique birds which lend themselves to exploring these questions (Figure 1A). Blue-eyed Shags are a monophyletic complex of 13 sister-species of marine cormorants living on the coasts and islands of, or adjacent to, the Southern Ocean, where they forage mainly on inshore benthic fish [20]–[22]. Different Blue-eyed Shag species look very much alike and display similar behavioural patterns [20], [23], [24]. Like other cormorant species, Blue-eyed Shags present a male-biased SSD [25]. Interestingly, they also show stereotyped sexual differences in foraging behaviour that appear to be characteristic of the group: sexual divergences in foraging rhythms, prey size and dive depth [26]–[38].

**Figure 2:**
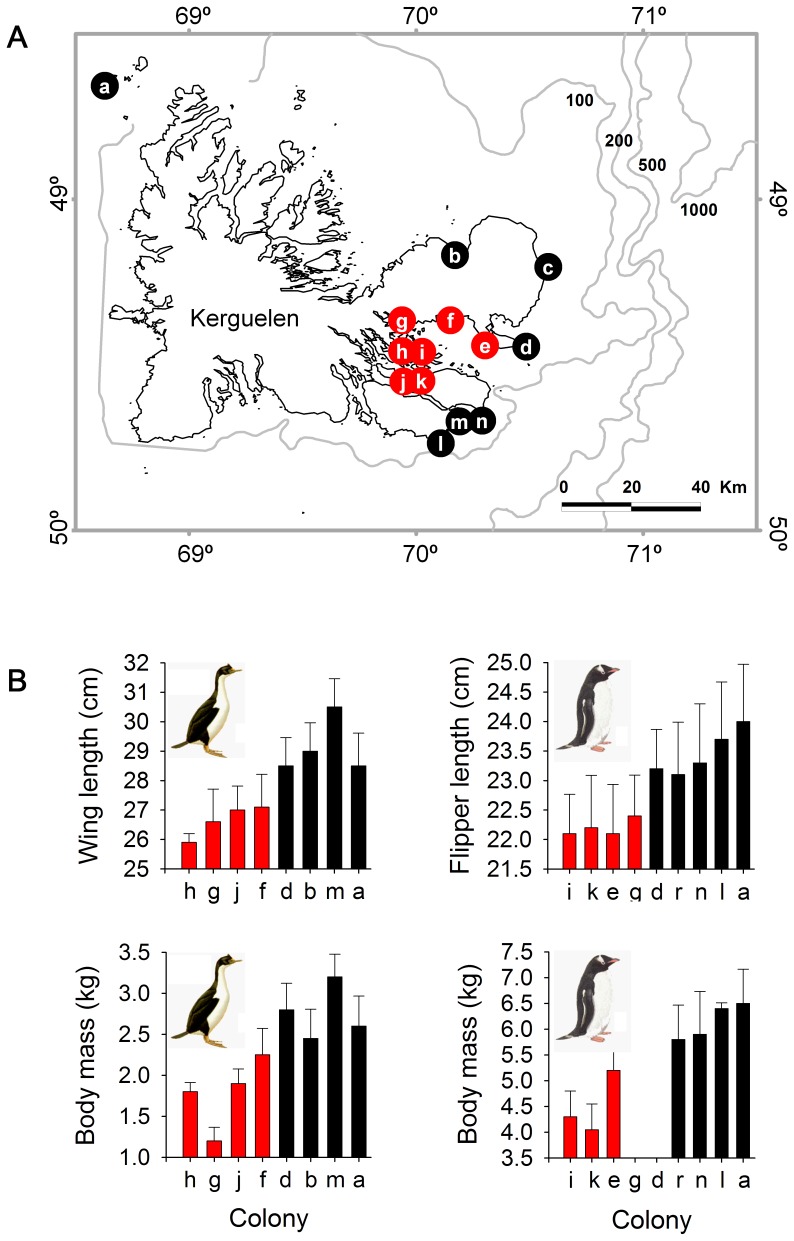
Geographic micro-variability of body size in the Kerguelen Shag and Kerguelen Gentoo Penguin. (A) Position of colonies at Kerguelen where measurements were performed on Kerguelen Shags and Gentoo Penguins. Colonies in red are inside a shallow and closed sea (the Golfe du Morbihan) and colonies in black are facing the open ocean. (B) Average body mass (±SD) and average wing/flipper length (±SD) of Kerguelen Shags (left) and Gentoo Penguins (right) at these colonies. Redrawn after [39].

We investigated the factors driving the evolution of body size in Blue-eyed Shags. For this, we first focused on the Kerguelen Shag *Phalacrocorax verrucosus* (Figure 1B). The Kerguelen Shag is an intriguing species because its average body mass (i.e. the average between mean female and mean male body masses) is known to vary by up to 77% between colonies only 35 km apart. Another inshore benthic forager, the sympatric Kerguelen Gentoo Penguin *Pygoscelis papua*, displays similar morphometric variability at a micro-geographical scale (Figure 2A and 2B) [39]. Four main factors can be advanced for this common pattern: sexual selection, developmental plasticity, temperature and prey patch depth.

### Sexual selection

Sexual selection is one of the most common forces cited for explaining patterns of variation in the body size of many vertebrate groups [19]. In theory, variation in sexual selection pressures can explain variation in average body size: male-male competition is thought to lead to an increase in female body size through sexual genetic correlations, though less so than in males [19]. This is the explanation for Rensch’s rule, which predicts that SSD should increase with average body size in species with male-biased SSD [40]. Rensch’s rule has shown strong consistency across bird taxa [41]. In cormorants, the study of sexual selection has received little attention (but see [42], [43]). Yet, its influence is highly probable in Blue-eyed Shags, either because of the agonistic interactions that occur between males during reproduction (*personal observation*), or simply because SSD is male-biased within the family [20], [25]. However, variation in sexual selection pressure is unlikely to explain, by itself, the disproportionate differences in average body size found between neighbouring colonies of Kerguelen Shags. The sympatric Gentoo Penguin is not related to the Kerguelen Shag and it has a different mating system [20], [44]. Yet, at Kerguelen this species presents a pattern of variation of average body size similar to the shag, with small-individual and large-individual colonies of both species located in the same areas of the archipelago respectively (Figure 2). Therefore, rather than a result of sexual selection, such a common pattern is best explained by the same ecological factor acting concurrently on both species.

### Developmental plasticity

A non-adaptive process involving geographic variability in food abundance during chick growth was hypothesized as a possible cause of body size variation in the Kerguelen Shag and Gentoo Penguin (developmental plasticity hypothesis [39]). Although poor provisioning may affect growth rate in some avian species, maturation is usually delayed and fledging body size is generally only partially affected [45]. Permanent stunting only exists in some extreme cases of nutritional stress and generally has severe consequences for fitness [45]. In cormorants, provisioning experiments in the Double-crested Cormorant *Phalacrocorax auritus* showed that, although chicks responded to food-shortage by allocating less energy to fatty tissues, muscles and visceral organs, their structural growth (skeleton) remained rigidly intact [46]. Therefore, although it is possible that growth nutritional stress may affect adult body size in Blue-eyed Shags or Gentoo Penguins to some extent, it is unlikely to explain the considerable differences observed between neighbouring colonies.

### Temperature

Bergmann’s rule predicts that endotherms should evolve larger body sizes in colder conditions, because heat loss rate decreases with increasing body size [47]. In theory, due to the higher heat absorbing properties of water relative to air, this rule should apply as much, or more, to diving endotherms as to their terrestrial counterparts. Sea water temperature was suggested as a possible driver of the evolution of body size in the Kerguelen Shag and Gentoo Penguin [39].

### Prey patch depth

Variation in biometry in these two species could be related to variation in diving adaptation according to locality [39], birds adapting locally to different distributions of benthic prey density with depth. In diving birds and mammals, where foraging consists of submerging in search of prey and surfacing regularly to reload oxygen reserves [48], there is a positive relationship across taxa between body size and dive capacity (e.g. [49]–[53]). This is due to bigger species having larger oxygen stores for a smaller mass-specific metabolic rate. Interestingly, studies have shown that the larger sex forages deeper than the smaller one in some species of whales, seals, penguins, boobies and cormorants (for a review, see [54], [55]). The allometry of dive capacity was hypothesized in some of these studies as the likely mechanism explaining such patterns and sexual niche divergence selection was proposed as a possible cause in some cases. In an article relating diving behaviour to body size [56], Mori concludes: “As optimal body size is related to prey depth, it follows that if prey distribution is bimodal in a habitat, two optimal body sizes can exist, which can induce body size dimorphism in a predator population. Thus, if the vertical distribution of prey in the water column is patchy, polymorphism in a predator population may be encouraged via habitat specialization” (p. 275). This statement was based on a modelling approach.

We studied two aspects of blue-eyed shag ecology pertinent to questions regarding size polymorphism. (1) We first described the morphology and diet of the Kerguelen Shag at different colonies and in both sexes. Since most piscivorous birds can be considered as gape-limited, bill morphology might be under selection for capturing fish of different sizes in the two sexes, as was suggested for two cormorant species from outside the Blue-eyed Shag complex [57]–[59]. To examine this, we studied the allometry of bill morphology and compared fish size between colonies and sexes. We also studied the foraging behaviour of the Kerguelen Shag at different colonies and in both sexes. (2) We then compared our findings with results obtained in the literature for Blue-eyed Shags from other localities and investigated the factors which could explain the variation of body size in this group of birds. We tested three hypotheses: (i) the consistency of Rensch’s rule across colonies (the effect of sexual selection), (ii) the effect of temperature on body size at colonies (Bergman’s rule) and (iii) the hypothesis of a diving adaptation at each colony. For hypothesis (iii), we tested whether dive depth depended on body size in breeding adults. Dive depth is a generally accepted proxy for depth of high prey density in many diving birds and mammals (e.g. [60]–[63]). In particular, we tested whether the effect of body size on dive depth or duration still held when accounting for colony and sex. We hypothesized that if the body size of each sex was subject to ecological selection–due to an adaptive advantage of a vertical partitioning of foraging depths between the sexes–SSD should reflect the extent of vertical partitioning and should vary from one colony to another in response to diverse situations of resource distribution and abundance.

## Methods

### Ethics statement

The study was performed with permission of the French Polar Institute (Institut Paul Emile Victor–IPEV), the organisation which oversees all research in the French sub-Antarctic and Antarctic districts. Field work was approved by the institute’s ethics committee and was conducted under IPEV research program number 394 (Diving Seabirds, coordinator: Charles-André Bost).

### Kerguelen Shags: morphology, diet and foraging behaviour

**Figure 3:**
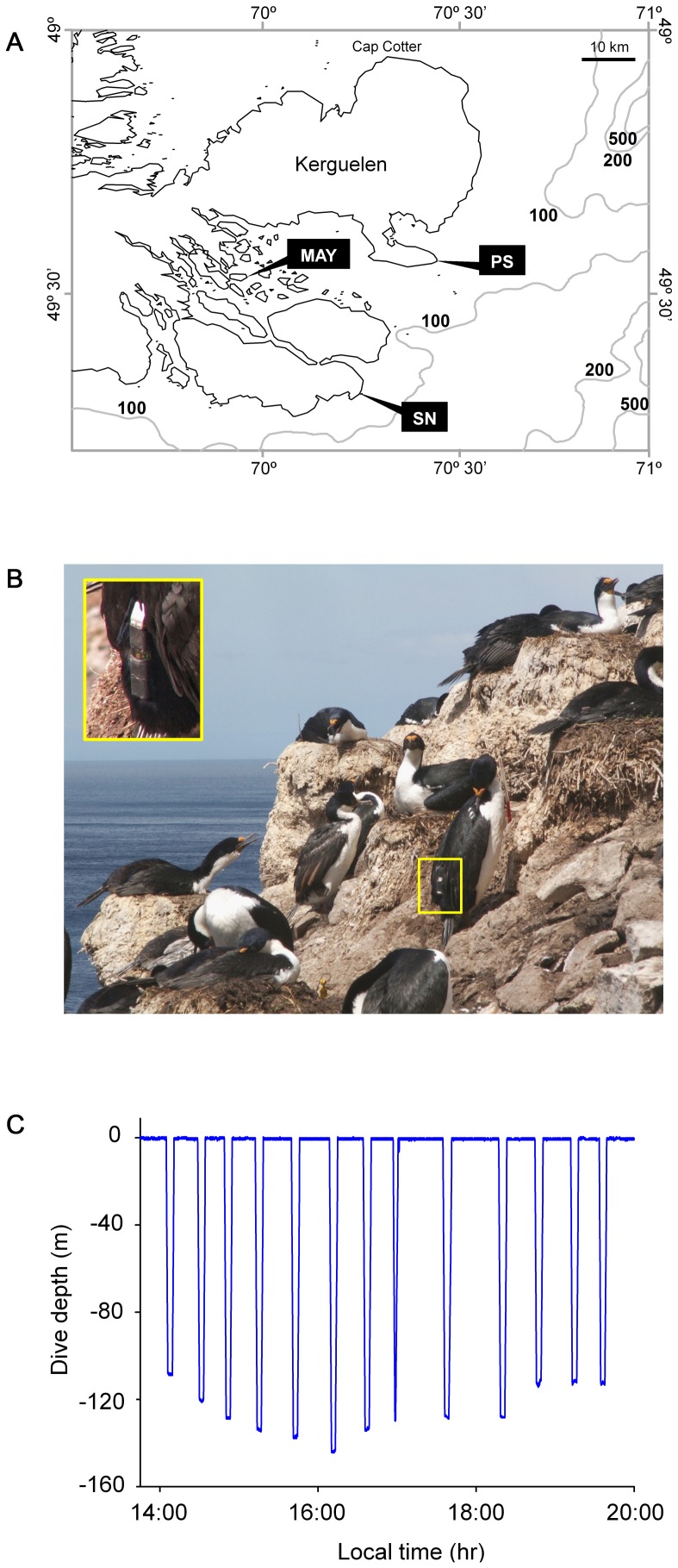
Position of study colonies of Kerguelen Shags and example of individual dive profile recorded by temperature-depth recorder. (A) Map of Kerguelen with the three colonies of Kerguelen Shags monitored in this study: Mayès (MAY), Pointe Suzanne (PS) and Sourcils Noirs (SN). (B) Bird equipped with a temperature-depth recorder (Sourcils Noirs colony). Photo: Timothée Cook. (C) Complete daily dive recording of a male bird from the Sourcils Noirs colony. Dive n° 6 reached a maximum depth of 144 m and dive n° 9 lasted 6.72 min.

The study was conducted during the Austral summer in the Kerguelen Archipelago. Three different colonies of breeding Kerguelen Shags were studied alternately over a period of 2–3 weeks each from December 2005 to March 2006. Each colony is separated from the next by 30–40 km and was set in a distinct oceanographic context (Figure 3A). The Mayès colony is located on an island at the far end of a large and sheltered bay (Golfe du Morbihan) where waters are rarely deeper than 50 m. The Pointe Suzanne colony faces the open ocean, with rapidly deepening offshore waters. The Sourcils Noirs colony also faces the open ocean, with an even steeper seafloor slope.

#### Morphology

Birds were caught during the day next to their nest, using a noose attached to the end of a telescopic fishing pole. They were then weighed and morphometric measurements were taken, including tarsus length, wing length, bill length (bill-tip to base of culmen), bill depth and width (at base of culmen) and bill length+head (from bill-tip to occiput). Individuals were sexed by comparing the two partners from a same nest: males are more aggressive at defending the nest and vocalize with a ‘honk’ (whereas the females vocalize with a ‘hiss’ [23]) and are larger [64], [65]. For 16 birds, uncertainty of sex led to further molecular sexing and results showed 100% agreement with the sex tentatively assigned in advance.

SSD was expressed as the sexual difference in the body trait divided by the female body trait. To compare body size between colonies and sexes, we performed a principal component analysis (PCA) on all body measurements, body mass excluded. This allowed for the exploration of differences in body size when accounting for individual differences in body condition. The first principal component axis (PC1) accounted for 78% of variance, and all original variables were positively correlated with it (all Pearson's product-moment correlation coefficients>0.80, all *P*<0.0001). We thus used PC1 scores as a synthetic measure of body size for comparing sexes and colonies. It should be noted that, overall, PC1 scores were highly correlated with body mass (Pearson's product-moment correlation coefficient = 0.90, *P*<0.0001). We used general linear models (GLMs) to test the effect of sex and colony (fixed effects and *a priori* interaction) on body size.

Finally, we tested whether bill size could be under differential selection for capturing fish of different sizes in each sex. For this, we studied the static allometry of bill size, i.e. we tested whether bill size increased more rapidly in one sex than in the other (Appendix S1a).

#### Diet

Wherever possible, stomach contents were collected through spontaneous regurgitation for diet analysis and blood and feathers were collected for isotope analysis (Appendix S1b). To investigate dietary differences between colonies and sexes, we compared the assemblage compositions of stomach contents using an analysis of similarity (ANOSIM) [66]. We used generalized linear mixed-effects models (GLMMs) to test the effect of sex and colony (fixed effects) on prey size. Diet sample number was set as a random effect because a same content will contain several prey items (repeated measures). We compared our results on sexual differences in prey size with other studies on Blue-eyed Shags (Appendix S2a).

#### Foraging behaviour

Study birds were captured at their nest and hooded during handling to minimize stress. Handling time was always <10 min. The data loggers were attached to the feathers of the bottom of the back, using Tesa^®^ tape (number 4651) secured with cyanoacrylate glue (Loctite^®^ 401). Data loggers were positioned on the back lengthwise relative to body axis (Figure 3B). Birds were equipped with electronic temperature-depth recorders for a period of 1–5 days. Recorders were streamlined MK9 (Wildlife Computers^®^) and M-190L-D2GT (Little Leonardo^®^) models. These measured 6.7×1.7×1.7 cm and 6.0×1.6 (diameter) cm and weighed 30 g and 16 g, respectively, representing 1–2% of body mass and ∼1–2% of cross-sectional area (based on a frontal area of 150 cm^2^ for a 2.5 kg Great Cormorant *Phalacrocorax carbo* calculated using software Flight 1.24 by CJ Pennycuick [67]). They were programmed to record depth and temperature every second with a precision of±0.5 m and±0.05 °C (MK9) and±0.1 m and±0.05 °C (M-190L-D2GT) (Figure 3C). Upon recapture, data loggers were retrieved and birds measured.

Dive recordings were analyzed with MultiTrace (Jensen Software Systems). Foraging trips were characterized using the temperature contrast between the cold seawater and the warmer air. Dives were considered as occurring when they were ≥1 m deep. For every dive we calculated dive duration and post-dive interval (defined as the surface post-dive recovery period between the end of one dive and the onset of the next). The bottom period was defined as starting and ending when the vertical transit rate of the bird became <0.25 m.s^−1^ (end of descent) and >0.25 m.s^−1^ (beginning of ascent) [68]. Dives were separated according to shape into two large categories: V-shaped dives and flat-bottom dives. V-shaped dives are theoretically associated with pelagic diving (within the water column), whereas flat-bottom dives are normally associated with benthic diving (on or near the seafloor) [69], [70]. In total, 45 birds were equipped for 2±1 days, producing a total of 3 months of individual temperature-depth recording comprising 204 foraging trips and 7159 dives.

We first examined whether sexual differences in foraging schedule and diving behaviour found at other colonies of Blue-eyed Shags (Appendix S2a) were also present in the Kerguelen Shag. We then used GLMMs to test the effect of sex and colony (fixed effects) on dive depth, dive duration and water temperature at the bottom of dives. Individual identity was always set as a random effect because a bird produced several dives (repeated measures). We also compared measures of dive capacity between sexes. In avian divers, the dive duration to post-dive interval ratio (dive to surface ratio) normally increases with dive depth and duration, reaches a peak when oxygen stores from the lung and air sacs are depleted, and decreases afterwards for deeper and longer dives [71], [72]. Dive efficiency (bottom duration/[dive duration+post−dive interval]) provides the proportion of time relative to the whole dive cycle that a benthic diver spends at the bottom of a dive, thus potentially foraging [73]. It is a correlate of dive to surface ratio because bottom duration and post-dive interval both increase with dive duration [72]. Dive efficiency and dive to surface ratio are thus expected to respond in a similar fashion. If a larger body size confers greater oxygen stores and a lower mass-specific metabolic rate, males are expected to dive for longer than females when dive depth is equal and, more importantly, to have a proportionately smaller post-dive interval (their dive to surface ratio and dive efficiency should both be greater). We only examined the effect of sex on these parameters at Mayès, because sexes overlapped in range of dive depth at this colony and were therefore comparable. We used GLMMs to test the effect of sex and dive depth (fixed effects and *a priori* interaction) on dive duration, dive to surface ratio and dive efficiency, including bird identity as a random effect to account for repeated individual dives. Finally, the relationship between dive to surface ratio and dive depth is shaped with a peak and a long right-skewed tail [71], [72]. Therefore, tests were only performed for depths greater than the peak, since the relationship becomes almost linear after this point, and differences in dive capacity are expected to be stronger at greater depths.

### Testing the factors driving the evolution of body size in Blue-eyed Shags

We tested the consistency of Rensch’s rule and the effect of temperature and food patch depth on body size in Blue-eyed Shags. For this, we collected all published data on the body size and dive capacity of individual Blue-eyed Shags across the Southern Ocean and the associated air and sea water temperature (for details on location and studies, see Figure 1B and Appendix S2b). Body mass was generally the only parameter available in the literature for describing body size. This was deemed sufficient however, as there is good correlation between body size and body mass (see Methods). Furthermore, the use of body mass enables comparison of results with previous studies on the allometry of dive capacity (e.g. [50], [52]). In this sense, body mass should be understood as a proxy for body size in this study.

#### Rensch’s rule

We collected data on body mass of Blue-eyed Shags from nine colonies, including the ones in this study. Using a linear regression, we investigated the relationship between SSD and mean body mass. We also tested the allometric plot of log_10_(female body mass) on log_10_(male body mass). The allometry is consistent with Rensch's rule if the slope of the regression is <1 [41].

#### Effect of temperature

Data were collected for Blue-eyed Shags from nine colonies, including the ones in this study. We used linear regressions to test the effect of air and sea water temperature on mean body size. Air temperature was calculated as the mean annual air temperature and sea water temperature was measured as the average sea surface temperature near the colony during breeding season.

#### Effect of prey patch depth

We collected data on the dive depth and duration of 110 individual Blue-eyed Shags from eight colonies, including those in this study (and also a value of average dive depth and duration for each sex from a ninth colony). We used maximum and mean values because both measures have been used before in the study of the allometry of diving capacity (e.g. [50], [52]). We first tested the effect of body mass on dive depth and duration using linear regressions. We then used GLMMs to test the effect of body mass (fixed effect) on dive depth and duration when accounting for sex (fixed effect) and colony (colony was set as a random effect because birds from different colonies may dive to entirely different depths due to different local ecological settings). We also tested whether there was an effect of SSD on mean sex difference in dive depth and duration using linear regressions. Finally, we examined the effect of dive depth on colony size using a linear regression. In most bird species, colony size is believed to be proportional to the amount of food available within foraging range [74], [75]. Hence, dive depth of birds from the largest colony should reflect depth of highest prey density between colonies.

### Softwares and statistics

Maps were drawn using ArcView 3.2 (Environmental Systems Research Institute Inc. 1999). All graphs and linear regressions were performed under Sigmaplot 10.0 (Systat Software Inc. 2002). Other statistical analyses were performed using R 2.15.0 software (R Development Core Team 2009). For GLMs and GLMMs, the significance of the factors was obtained after performing an ANOVA on the models. Statistical significance was accepted at the α≤0.05 level. Means are presented±SE.

## Results

### Geographical and sexual variation in morphology, diet and foraging behaviour of Kerguelen Shags

#### Morphology

Birds from Mayès were significantly smaller than birds from Pointe Suzanne, who were in turn significantly smaller than birds from Sourcils Noirs (*F*_2,102_ = 280.27, *P*<0.0001). Within each colony, males were significantly larger than females (*F*_1,102_ = 202.17, *P*<0.0001) and the relative difference between the sexes (SSD) varied significantly from one colony to another (*F*_2,102_ = 3.31, *P* = 0.040) (table 1). Study of the static allometry of bill size did not support the hypothesis that bill was under differential selection as a means for capturing fish of different sizes in each sex (Appendix S1a).

**Table 1:** Morphological differences between sexes and colonies of Kerguelen Shags. SSD is expressed as the sexual difference in the body trait divided by the female body trait.

	Mayès			Pointe Suzanne			Sourcils Noirs		
	Females	Males	SSD	Females	Males	SSD	Females	Males	SSD
Parameter	(*N* = 15)	(*N* = 12)	(%)	(*N* = 20)	(*N* = 21)	(%)	(*N* = 22)	(*N* = 18)	(%)
Body mass (g)	1,552±79	1,842±137	18.7	1,994±160	2,614±326	31.1	2,800±154	3,218±305	14.9
Bill length (mm)	45.6±1.7	49.8±2.3	9.2	49.1±1.7	53.5±2.2	9.2	52.3±2.9	54.2±2.3	3.6
Bill depth (mm)	12.1±0.6	13.3±0.6	9.9	13.4±0.7	15.6±1.3	16.4	15.3±1.6	16.8±1.4	9.8
Bill width (mm)	10.4±0.4	11.4±0.5	9.7	11.4±0.76	13.0±1.4	14.7	11.9±1.0	13.5±1.4	13.4
Head + bill length (mm)	113.1±2.4	119.8±2.9	5.9	121.6±2.5	130.8±3.6	7.6	129.6±3.4	136.1±4.1	5.0
Tarsus length (mm)	56.0±1.9	58.5±1.7	4.4	60.9±1.4	65.9±2.7	8.2	67.7±3.3	70.5±2.2	4.1
Wing length (mm)	254.9±5.9	271.3±5.0	6.4	272.8±5.3	291±7.0	6.7	290.4±6.3	307.7±9.3	5.9

#### Diet

Stomach content analysis revealed that diet at all three localities was composed of 95% benthic fish (849 fish items identified, Appendix S1b). The ANOSIM showed a difference in the relative abundance of each prey species between colonies (*R* = 0.25, *P*<0.001). However, there was no difference in diet composition between sexes at Pointe Suzanne (*R* = 0.04, *P* = 0.236) or at Sourcils Noirs (*R* = 0.07, *P* = 0.199), the colonies where stomach contents were collected from individuals of known sex. Combining all fish species, fish captured by birds at Pointe Suzanne were not significantly different in standard length from fish captured by birds at Sourcils Noirs (*F*_1,332_ = 2.29, *P* = 0.131). When accounting for colony, there was a trend for males to catch longer fish than females (*F*_1,37_ = 3.73, *P* = 0.061) (Appendix S1b). By testing only for the dominant species at Pointe Suzanne (*Notothenia cyanobrancha*: 74%) and at Sourcils Noirs (*Lepidonotothen mizops*: 73%), there was still no difference in fish standard length between colonies (*F*_1,259_ = 1.89, *P* = 0.170), but fish ingested by males were significantly longer than fish ingested by females at Pointe Suzanne (males: 9.0±5.3 cm; females: 4.9±3.4 cm) and at Sourcils Noirs (males: 9.3±1.7 cm; females: 7.2±2.1 cm) (*F*_1,33_ = 6.63, *P* = 0.014). These results are in accordance with those obtained from the isotope analysis (Appendix S1b). Sexual differences in prey size concur with results obtained in studies on other Blue-eyed Shags (Appendix S2a).

#### Foraging behaviour

**Figure 4:**
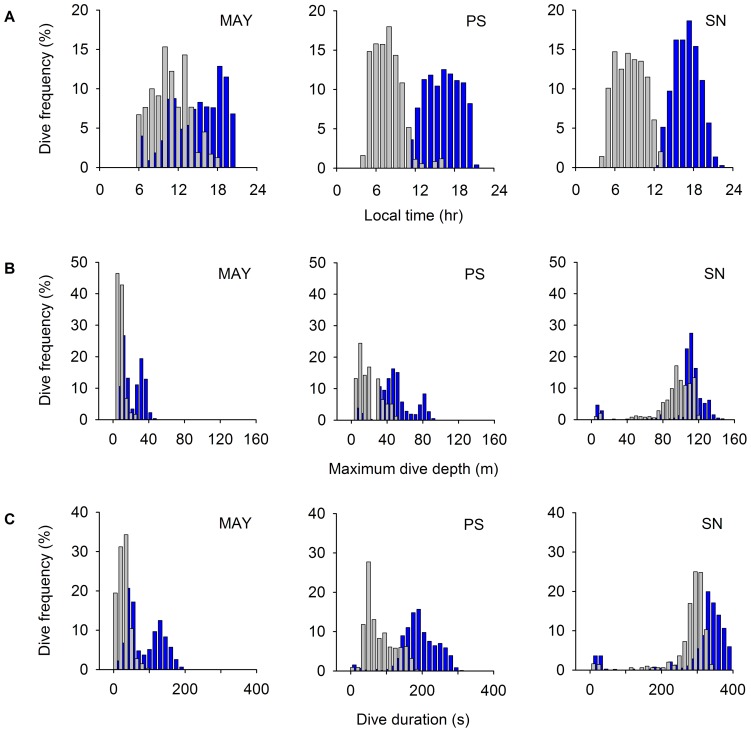
Sexual differences in diving behaviour in the Kerguelen Shag. Distribution of dives in relation to (A) time of day, (B) maximum dive depth and (C) dive duration. Colonies are Mayès (MAY), Pointe Suzanne (PS) and Sourcils Noirs (SN). Females are in grey, males in blue.

**Figure 5:**
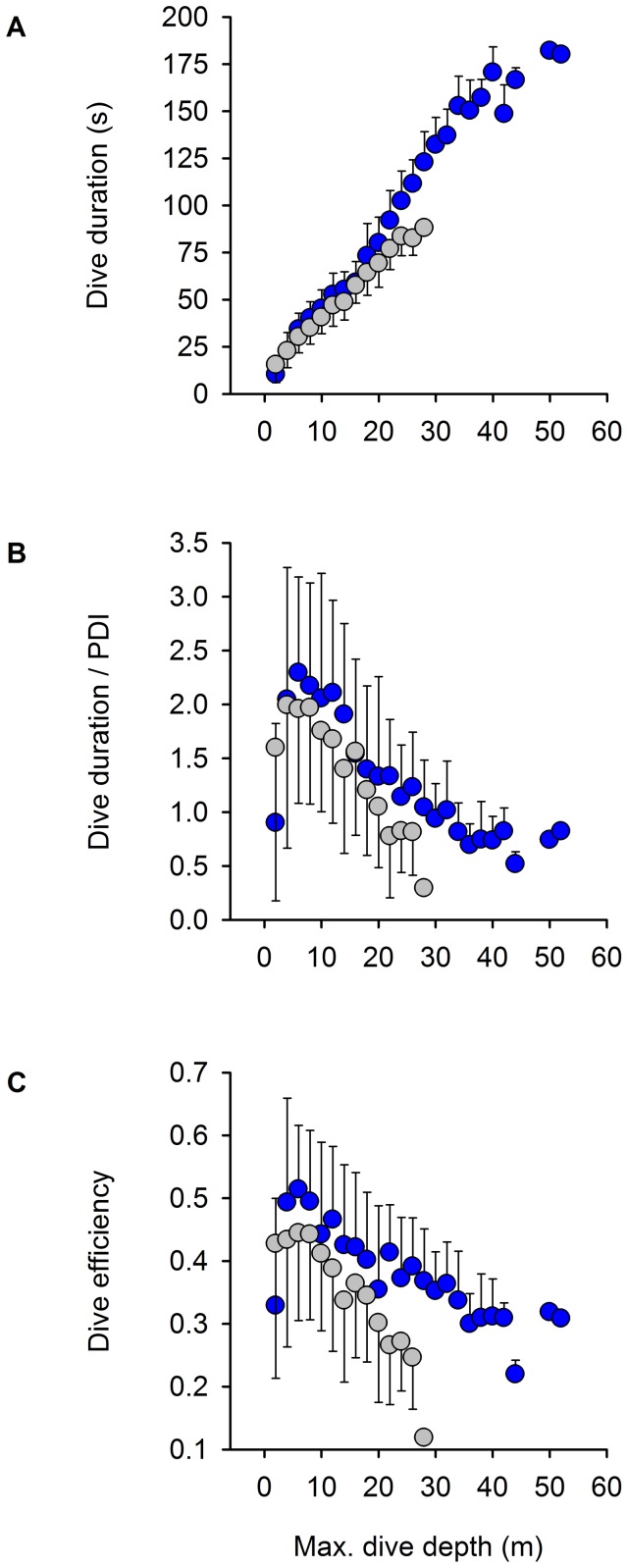
Differential diving capacity between sexes according to depth in the Kerguelen Shag. Effect of dive depth on (A) dive duration, (B) dive to surface ratio and (C) dive efficiency in birds from the Mayès colony. PDI is the post-dive interval and dive efficiency is calculated as bottom duration/(dive duration+post−dive interval). Females are in grey, males in blue.

Birds foraged exclusively during daylight, the earliest commuting to foraging grounds just before sunrise and the latest returning to the colony just after sunset (for details on time-budget and dive parameters, see Appendix S1c). Dive profiles were dominantly flat-bottomed, suggesting benthic diving, in agreement with the dietary analysis. Birds from Mayès, Pointe Suzanne and Sourcils Noirs dived in shallow, intermediate and deep waters respectively (*F*_2,41_ = 224.77, *P*<0.0001), and mean water temperature at the bottom of dives decreased from Mayès to Pointe Suzanne and from Pointe Suzanne to Sourcils Noirs (*F*_2,40_ = 180.00, *P*<0.0001). Sex-specific patterns of foraging behaviour characteristic of other Blue-eyed Shags (Appendix S2a) were also found in the Kerguelen Shag. At all three colonies, females dived primarily in the morning and males in the afternoon (Figure 4A), and female dives were on average shallower (*F*_1,41_ = 22.11, *P*<0.0001) (Figure 4B) and shorter than male dives (*F*_1,41_ = 23.64, *P*<0.0001) (Figure 4C). At Mayès, females dived mostly in waters <15 m deep, whereas male dives had a bimodal distribution, with a peak at 10 m and a peak at 35 m. Segregation was strongest at Pointe Suzanne, where females were restricted to depths ∼20 m, while males foraged in waters ∼50 m. At Sourcils Noirs, all birds foraged deep, females diving to ∼90 m and males to ∼100 m. Mean water temperature at the bottom of dives was warmer for females than for males (*F*_1,40_ = 5.21, *P* = 0.028). At Mayès, both sexes reached the peak in dive to surface ratio for dives of a maximum depth of 5–10 m and lasting 35–50 s (estimated visually; Figure 5A and 5B). Dive efficiency followed the same pattern (Figure 5C). Results showed no apparent sex difference in the position of this peak for either parameter. For depths greater than the peak (arbitrarily positioned at 7.5 m), dive duration (*F*_1,1572_ = 165.48, *P*<0.0001), dive to surface ratio (*F*_1,1571_ = 10.31, *P* = 0.001) and dive efficiency (*F*_1,1551_ = 23.74, *P*<0.0001) increased more quickly in males than in females when accounting for dive depth.

### Factors driving the evolution of body size in Blue-eyed Shags

#### Rensch’s rule

The relationship between SSD and body size was not consistent with Rensch’s rule (Appendix S2c).

#### Temperature

Mean colony body mass did not depend on mean annual air temperature or on sea surface temperature during the breeding season (Appendix S2d).

#### Prey patch depth

**Figure 6:**
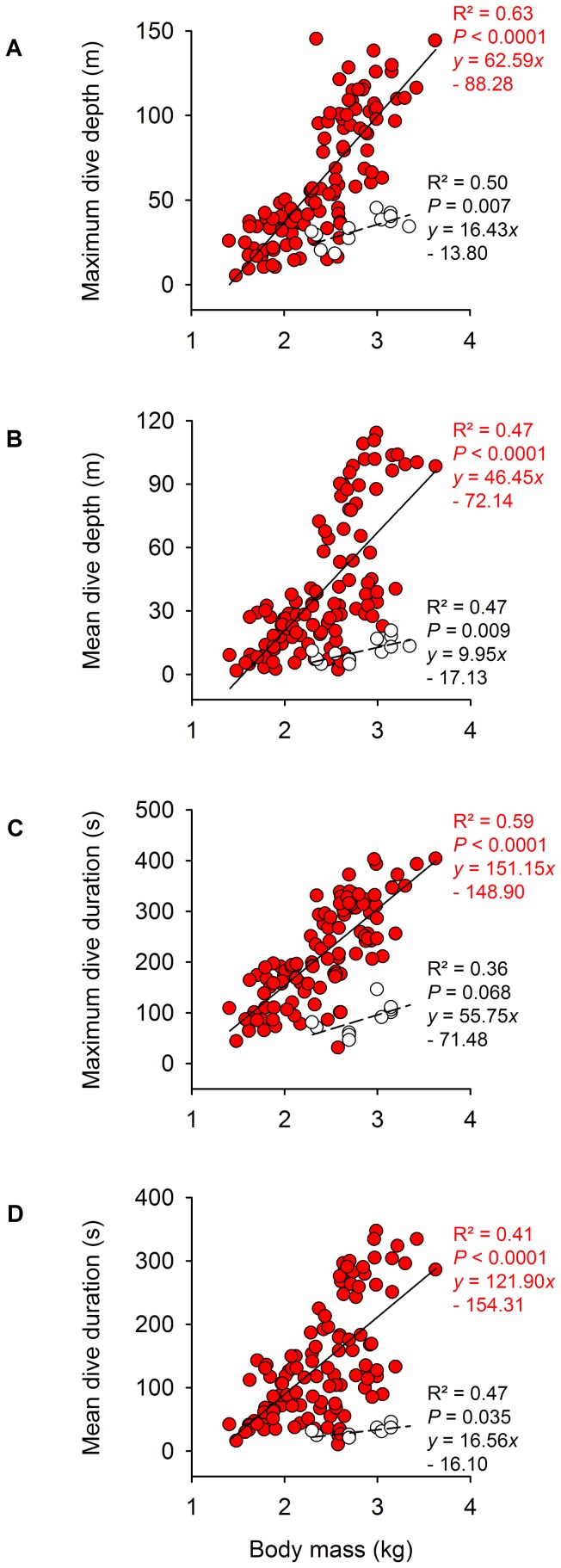
Relationship between body mass and diving capacity in Blue-eyed Shags. Effect of body mass on (A) maximum dive depth, (B) mean dive depth, (C) maximum dive duration and (D) mean dive duration for 110 Blue-eyed Shag individuals from eight colonies. For comparison, data is provided for 13 birds from a species outside the Blue-eyed Shag complex (white circles, dashed line), the Japanese Cormorant *Phalacrocorax capillatus* (data from [76]).

**Figure 7:**
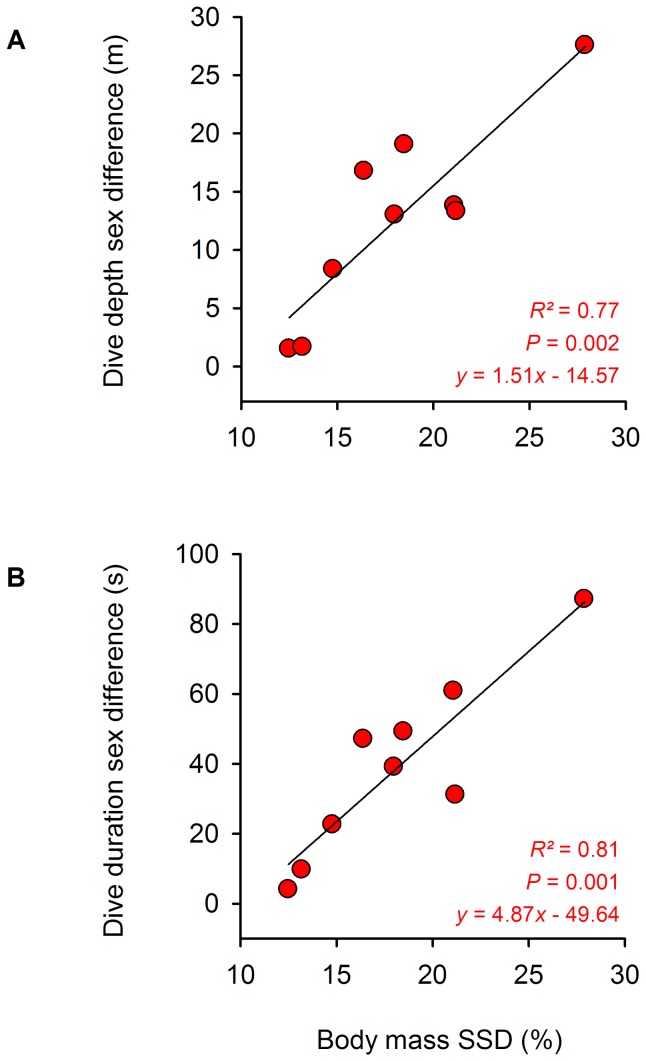
Relationship between SSD and sexual differences in diving capacity in Blue-eyed Shags. Effect of SSD on mean sex difference in (A) dive depth and (B) dive duration for nine study colonies of Blue-eyed Shags. For each colony, mean sex difference in dive capacity is calculated as the difference, between the sexes, in the average of individual means.

Maximum dive depth, mean dive depth, maximum dive duration and mean dive duration all depended directly on body mass (Figure 6A, 6B, 6C and 6D). This effect held for maximum dive depth (*F*_1,100_ = 97.84, *P*<0.0001), mean dive depth (*F*_1,100_ = 43.89, *P*<0.0001), maximum dive duration (*F*_1,100_ = 91.10, *P*<0.0001) and mean dive duration (*F*_1,100_ = 35.63, *P*<0.0001), when accounting for colony and sex (see also Appendix S2e). In other words, sexual differences in dive capacity were related to sexual differences in body size, not to sex *per se*. There was an effect of SSD on the sexual difference in mean dive depth and duration (Figure 7A and 7B). Colony size depended on dive depth at colonies where birds fed on notothenioid fish, suggesting that food was more abundant at greater depths (Appendix S2f).

## Discussion

Results on the morphology of Blue-eyed Shags did not support the sexual selection hypothesis (inconsistency of Rensch’s rule). This corroborates earlier observations of patterns of body size variation across Kerguelen in the sympatric Kerguelen Shag and Gentoo Penguin (Figure 2), suggesting that the selective pressure driving such patterns is an ecological factor acting concurrently on both species. It suggests furthermore that sexual selection is unlikely to explain, by itself, the patterns of variation of SSD reported in Blue-eyed Shags in this study. Two ecological factors–air temperature and sea water temperature–had no effect on mean body mass, ruling out Bergmann’s rule as a likely driver for the evolution of body size in this group. The study of foraging behaviour, however, revealed that mean dive depth was explained by mean body mass in Blue-eyed Shags, supporting the hypothesis of a diving adaptation at each colony. At all colonies, sexes segregated temporally and vertically in diving behaviour. Since Blue-eyed Shags are benthic divers, the corollary of a vertical partitioning of foraging depths is a spatial partitioning of foraging grounds between sexes, something which has recently been confirmed with tracking devices [37], [38]. Furthermore, sexual differences in dive depth reflected the level of SSD at each colony, suggesting that sexual niche divergence could have an adaptive advantage. We next discuss the mechanisms by which local variations in prey species and prey patch depth and distribution could explain the evolution of body size in this group.

### Role of prey patch depth in the selection of body size

Preliminary results show that dive capacity is also explained by mean body size in Gentoo Penguins from Kerguelen [77]. This suggests that the similar geographical variation of size observed in Kerguelen Shags and Kerguelen Gentoo Penguins (Figure 2) could be the result of a common diving adaptation to foraging off resources located at different depths according to the colony. Interestingly, both species happen to prey on several identical benthic fish species [78]. The difference in diet of shags between nearby colonies does support the assumption that accessible benthic prey distribution and abundance are patchy. At Kerguelen, for example, prey species differ from one colony to the next, probably reflecting entirely different local ecological conditions, as witnessed by the different isotope signatures of bird blood and feathers and sea water temperatures between colonies. At two colonies, we found that 75% of prey items comprised one fish species. *Notothenia cyanobrancha* and *Lepidonotothen mizops* dominated at Pointe Suzanne and Sourcils Noirs respectively. Unsurprisingly, *N. cyanobrancha* prefers shallow and calm waters and the proximity of kelp beds [79] and at Pointe Suzanne conditions are relatively sheltered, with vast kelp forests, and birds dived in shallow and intermediate waters in this area. On the other hand, *L. mizops* prefers waters >20 m deep [79]. At Sourcils Noirs, conditions are exposed, with cold waters and a steep seafloor slope, and birds dived deeply in this area. Further evidence for prey patchiness can be found in individual bird behaviour. Study of the diving behaviour of individual Crozet Shags *Phalacrocorax melanogenis* over five consecutive days [80] showed that birds consistently used the same range of dive depths and that distribution of dive depth was close to normal (Appendix S2e). This not only suggests that mean values of dive depth or duration can be adequate indicators of individual dive preference, but also that repetitive individual dive habits could point to the existence of food patches which are localised and predictable in time and space.

Hence, for the Kerguelen Shag, as for other Blue-eyed Shags, patchiness of benthic resources means that food can have a shallow or deep distribution, depending on the colony. Deep diving is a costly behaviour, because the time for surface recovery increases faster than diving time as dive depth increases [81]. Birds are consequently expected to adjust their behaviour to optimise the time spent underwater [72], [82] and to adapt, i.e. to develop an optimal body size [56]. In eco-physiological terms, the optimal body size for diving to a particular depth is the one that yields important return rates in time and energy-budget. Dive efficiency refers to the time spent at the bottom of a dive (thus foraging) relative to the time invested in the overall activity of diving. For all birds, the highest values of dive to surface ratio and dive efficiency were found for dives 5–10 m deep lasting 35–60 s (Figure 5B and 5C). This is because the uptake rate of oxygen in the birds is fast during the post-dive intervals of these dives which deplete the respiratory tract oxygen stores (‘optimal breathing’) [72]. For deeper, therefore longer dives, efficiency decreases because the uptake rate of oxygen into the bird is slowed by the long replenishment of the highly depleted blood haemoglobin and skeletal muscle myoglobin stores [71]. For the latter dives, a bigger body would be expected to provide a higher efficiency than a smaller one, because it should use less oxygen per unit of mass while having larger oxygen stores [83]. This was the case in the Kerguelen Shag, where smaller birds (females) from Mayès had a dive efficiency of 0.25 for dives 25 m deep, while larger birds (males) from the same colony had almost double efficiency (0.40) at similar depth (Figure 5B and 5C). In other words, if prey is only abundant in deep waters, a larger individual can be expected to exploit these resources more efficiently, which should translate into increased fitness and selection over generations for increased body size.

**Figure 8:**
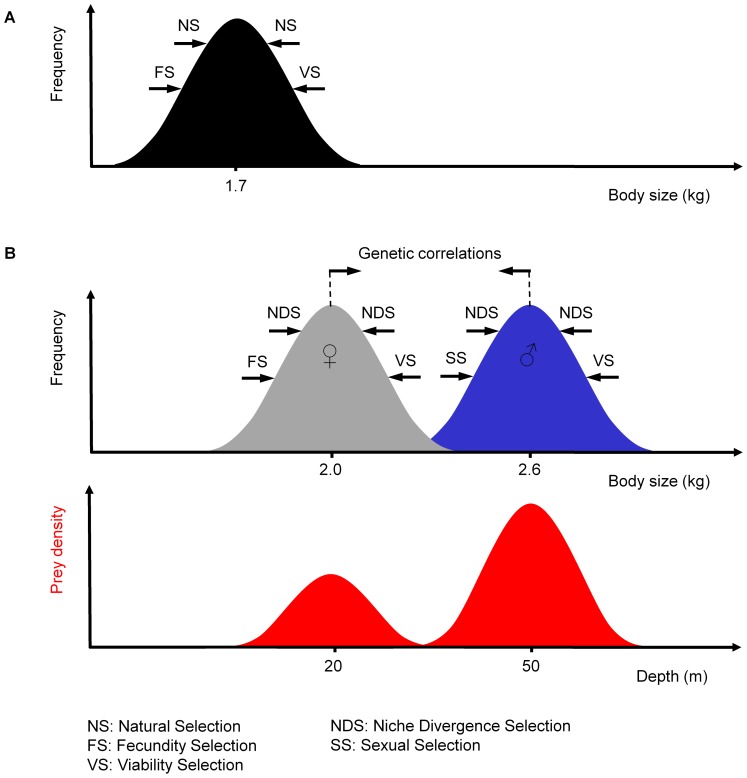
Selective forces influencing the evolution of body size in Blue-eyed Shags. (A) Theoretical situation with uniform prey density distribution with depth and absence of sexual selection. Under the influence of natural selection, body size is monomorphic and stabilizes around 1.7 kg (value derived from regressions in Figure 6B and 6D). Birds have the optimal size for carrying out dives with the highest efficiency (shallow dives 5–10 m deep: ‘optimal breathing’). A bigger body is also maximally efficient at such depths, but is counter-selected by viability selection. (B) Present situation with a heterogeneous prey density distribution with depth and sexual selection. Under the influence of sexual niche divergence selection, the body size of one or of both sexes stabilizes around a value that is optimal for exploiting a specific prey patch (actual examples of body size and depth measured for birds from the Pointe Suzanne colony at Kerguelen). Fecundity selection is indicated in both situations. However, because of the phylogenetic constraints for brood size and the high cost of rearing progeny in altricial birds, we considered fecundity selection to have a relatively limited influence on the present evolution of body size and SSD in Blue-eyed Shags. Drawn using concepts found in [84].

While small individuals are restricted to shallow waters, large ones can theoretically forage both in shallow and deep waters with adequate efficiency. Arguably, a single morph–a very large body–would seem like the most advantageous outcome of evolution at any Blue-eyed Shag colony, allowing birds access to the largest possible range of depths and prey patches. However, unless very abundant resources are to be found in deep waters, a large morph should be counter-selected due to the higher costs of being large, which involve longer growth periods, reduced agility or expensive maintenance (viability selection [84]). Furthermore, sexual dimorphism (male-biased SSD) in Blue-eyed Shags is obligate, due to the influence of sexual selection. This pre-existing SSD could be exaggerated by sexual niche divergence to various degrees depending on the colony and the vertical distribution of benthic resources nearby. Males, because they can dive deep, could be selected for increased size if even deeper, more offshore waters, hold considerably higher densities of resource. The genetic correlation between the sexes means that, over the generations, females would also increase in size. The resulting size selected for each sex could depend on several evolutionary forces acting concurrently, sometimes synergistically, sometimes agonistically (Figure 8).

It is reasonable to assume that the main object of a sexual niche divergence selection in Blue-eyed Shags would be overall body size (and concurrently body mass), since this is the character which provides the two sexes with differential dive capacities through its influence on oxygen stores and metabolism [83]. Study of the static allometry of bill morphology in the Kerguelen Shag did not support the hypothesis that bill shape and size could be under sexual niche divergence selection for capturing prey of different sizes (Appendix S1a). Furthermore, although female Kerguelen Shags ate smaller fish on average than males, they apparently had no problem eating fish that were as big as the largest caught by males (Appendix S1b). If gape-limitation determines sexual differences in prey size, why would females from Sourcils Noirs–which are of similar size as males from Pointe Suzanne–ingest fish that are smaller than those caught by the latter? Considering the size of the dominant fish species found in the stomach contents at Kerguelen, the fish captured by females corresponded primarily to individuals from the recently-recruited post-larval cohort, while the fish captured by males belonged to a previous cohort, or to older fish (>1 year old [79]). At sea, these two cohorts probably form two contiguous or separate patches, with the younger one closer inshore because shallower waters offer better protection from predators [79]. Although it seems more likely that each sex would target a prey patch (therefore a depth zone) rather than a prey size, the possibility of subtle selection processes also occurring differentially on the bill morphology of each sex in relation to the capturing and handling of fish of different sizes cannot be excluded. Examining this will necessitate studying the detailed functional anatomy of bones and muscles of head and bill in each sex [85]. Even so, it might remain difficult to draw a conclusion, as sexual selection in male-male combat operates not only on overall body size, but also on shape and size of head and bill [15].

### A scenario for the evolution of body size in Blue-eyed Shags

Although the phylogeny of this group is still incompletely known, molecular work suggests that Blue-eyed Shags are a monophyletic and recently diversified clade (Kennedy et al. 2000), possibly originating some 500,000 years ago from the Southern tip of South America and colonizing the Southern Ocean westwards, following the dominant westerly winds (Martyn Kennedy, *personal communication*). During this period, Blue-eyed Shags adapted to diving in cold water and specialized in feeding mostly off benthic fish (often notothenioids). Within the distribution range of this group, regional oceanographic conditions and micro-variations in the ecology of the marine habitat, could have determined the position and depth of predictable and abundant prey patches. Over time, this could have selected for variation in mean shag body size between localities. Concurrently, the male-biased SSD of Blue-eyed Shags evolved under sexual selection could have been exaggerated through sexual niche divergence selection because the distribution of prey would also be heterogeneous at fine spatial scale, with several patches of highest density occurring close to each colony, often at different depths. Interestingly, when extrapolating the regression between SSD and sexual difference in dive depth to a 0 m depth difference, SSD falls to 11.5% (Figure 7A), suggesting that dive capacity may diverge significantly only above such a difference in body size. One might speculate that, if a 0 m difference represented the absence of sexual niche divergence selection in Blue-eyed Shags, the remaining 11.5% of SSD could be attributed to sexual selection.

For this scenario to reflect evolution, several major assumptions must be made. (1) Some degree of genetic isolation must exist at each colony for adaptive processes to take place. This implies first that colonies are long-lived. Colonies are expected to survive if locally abundant prey patches remain stable over time. Predictability is supported by the fact that the marine environment has some immutable physical features. At Kerguelen for example, fish larvae are transported and recruited in predictable areas because of currents and underwater topography [86]. Bird nests measuring over 4 m in height (*personal observation*) are witness to the fact that some colonies have been occupied for several hundred years at least. Genetic isolation also implies that birds are highly philopatric. Results from a two-year study at one Kerguelen colony suggest this could be the case: around 70% of birds that were seen breeding at the colony the first year were seen breeding there the second (a figure that does not account for annual adult mortality, which should be around 20% [87]) and 96% of these came back to the exact same nest (*unpublished data*). Finally, the values of mean body size measured at Kerguelen were identical to those recorded 15 years earlier for the same colonies [39], suggesting that the selective pressures acting at that time are still in place today. (2) Variation in body size must be, at least in part, genetically determined. Although the mechanisms influencing body size are complex, studies have shown that adult size in birds is highly heritable [88]. (3) The ecological setting must be favourable to a selection of body size.

A favourable ecological setting would be one of limited and patchy food resources which would intensify local specialisation. The inshore benthic fish community of the Southern Ocean might be the perfect place for such conditions. A low prey abundance (compared to the pelagic zone) and a restricted production area (surface area of the coastal shelf adjacent to the colony) on one hand, combined with the fact that birds have a limited dive depth and foraging range on the other, may explain why there are so few Blue-eyed Shags compared to other marine top-predators in this area of the world. Blue-eyed Shags never number more than a few hundred individuals at any one locality, whereas colonies of pelagic foraging penguins are often tens or even hundreds of thousands strong [20], [44]. It may also explain why they display such extreme dive capacities compared to other cormorant species of similar size range (Figure 6), diving to great depths to find adequate patches of food. The positive relationship between colony size and dive depth suggests that food abundance increases with depth across colonies, although this seems to apply only to localities where birds feed predominantly on notothenioids (deeper diving would provide more food only if deeper patches were to be found within reasonable foraging distance from the colony). At the same time, fish size also appears to increase with depth (see above). Resources may be further limited through local depletion during the breeding season [89], a period when parental care may be very demanding and birds must be efficient foragers. In particular, species which depend only upon benthic prey would be more readily concerned by prey depleted halos around colonies than species which also depend on pelagic prey [37], and sexual partitioning of dive depths would decrease intra-specific competition. It is unclear what advantage the sexual partitioning of foraging schedule (Figure 4A) brings in this context. Hypotheses for this behaviour include decreased risk of chick starvation, synchronization of the activity of at least one of the two sexes with the activity of its favourite prey, and increased foraging efficiency of the breeding pair [26], [31], [35]. In short, there might be other ecological benefits to sexual niche divergence than simply reducing directly intra-specific competition (for a review, see [7]). If differences in foraging efficiency increase the combined niche breadth of the breeding pair, sexes need not be in competition. In ‘cooperation in exploitation’, provisioning efficiency–therefore fitness–is enhanced for pairs where niches are dimorphic, because a breeding pair will maximize its collective harvest. Whatever the precise mechanisms involved, they are probably numerous and overlapping. Studying foraging behaviour of male and female Blue-eyed Shags outside of the breeding season should reveal if niche partitioning in Blue-eyed Shags is linked to breeding constraints. In parallel, studying breeding success of pairs according to colony could inform on the fitness of individual males versus females relative to body size. Finally, at-sea surveys could inform on prey availability with depth, although this might likely prove logistically difficult.

## Conclusion

Studying the evolution of body size is rendered difficult by the existence of numerous possible selection pressures, sometimes acting simultaneously and interacting. A larger body size may be advantageous to exploit large food items, fight off predators, monopolize resources or females, survive in cold conditions and increase fecundity [12], [13], [47], [90]. A smaller body size may be advantageous to exploit small food items, reduce growth periods, lower maintenance costs, increase foraging agility or sexual displays and increase survival in warm conditions [47], [91], [92].

Effectively teasing apart the relative contribution of each parameter means studying all factors of the equation. The study of resource polymorphism, in particular, deserves attention, as it probably occurs more often than is generally appreciated despite being an important first step to population divergence or even to speciation [4]. Frequently, researchers interested in the evolution of SSD focus on the study of sexual selection because it is relatively parsimonious and easy to test [7]. Yet, selection for SSD through sexual niche divergence should be relatively common [8], albeit challenging to detect [7]. The difficulty in identifying the mechanisms underlying such processes should not deter researchers from addressing these questions. In this paper, we suggest that trophic resources may select for important geographic micro-variability in body size and SSD of endotherms. Although Blue-eyed Shags represent a powerful study model for exploring these questions, the principles guiding the allometry of diving capacity are universal. Situations of selection for polymorphism among populations and of sexual niche divergence selection could occur in a diverse range of diving birds and mammals.

## Supporting Information

Appendix S1 **Study of the Kerguelen Shag: (a) measure of the static allometry of bill size, (b) results from dietary studies and (c) details on foraging behaviour.**
(PDF)

Appendix S2 **Literature review on Blue-eyed Shags: (a) sex-specific foraging behaviour, (b) details of studies, (c) test of Rensch’s rule, (d) effect of temperature on dive capacity, (e) effect of body mass and sex on dive capacity and (f) effect of dive depth on colony size.**
(PDF)
