# Supplementary material for: Can Foraging Ecology Drive the Evolution of Body Size in a Diving Endotherm?
Source: PLoS One. 2013 Feb 7;8(2):e56297. doi: 10.1371/journal.pone.0056297 (PMC3567052; doi:10.1371/journal.pone.0056297)
Supplement: Appendix S1 — Study of the Kerguelen Shag: (a) measure of the static allometry of bill size, (b) results from dietary studies and (c) details on foraging behaviour. (PDF) [file pone.0056297.s001.pdf]

Appendix S1. Study of the Kerguelen Shag: (a) measure of the static allometry of bill size, (b) results from dietary studies and (c) details on foraging behaviour.

(a) Static allometry of bill size

To examine how bill characteristics change as body size increases with sex and colony, a principal component analysis (PCA) was built on tarsus and wing length. The first principal component axis (PC1) accounted for 92% of variance, and all original variables were positively correlated with it (all Pearson's product-moment correlation coefficients > 0.97, all $P < 0.0001$). Because this PCA was not calculated using bill measurements, PC1 scores could be used as synthetic measurements of body size for studying the static allometry of bill size in the Kerguelen Shag (Smith 1990).

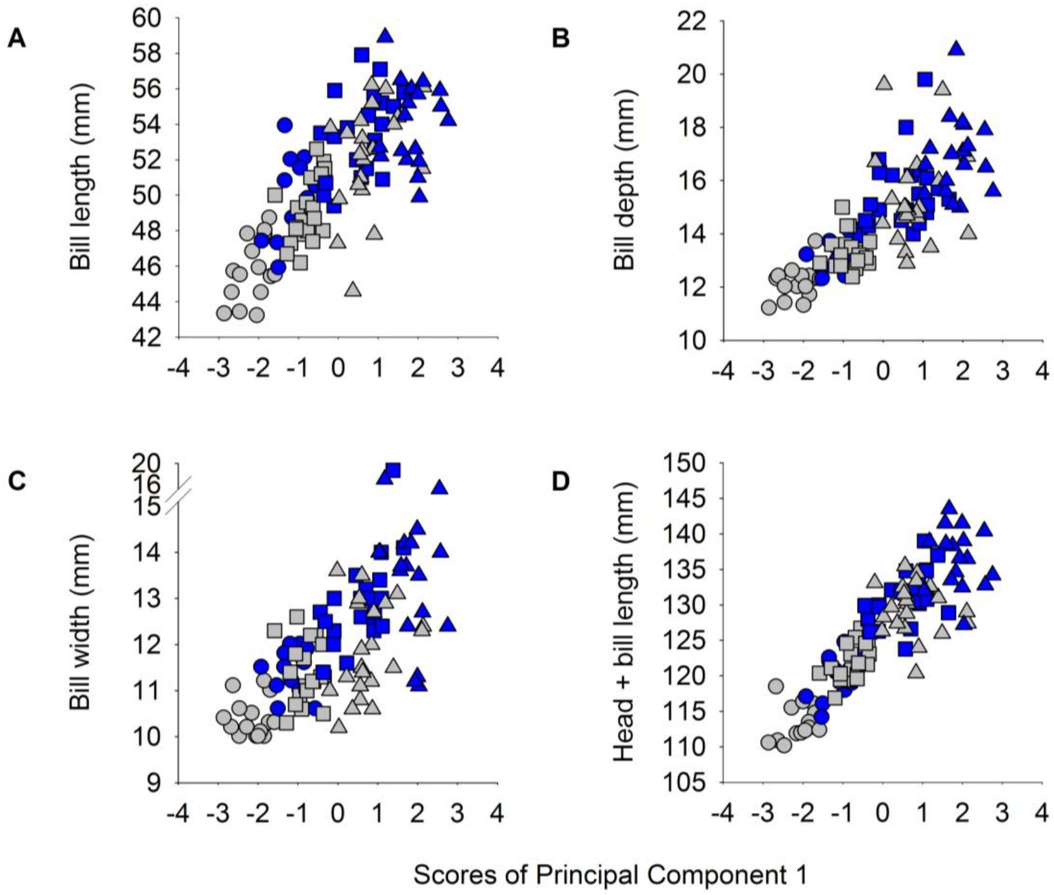

Figure A. Relationship between (A) bill length, (B) bill depth, (C) bill width, (D) head + bill length and the PC1 scores of the PCA performed on tarsus and wing length in the Kerguelen Shag. Individuals are from the Mayès (circles), the Pointe Suzanne (squares), and the Sourcils Noirs (triangles) colonies (females in grey, males in blue).

To test whether bill size increased more rapidly in one sex than in the other, when accounting for body size and colony, we constructed a generalized linear mixed-effects model with PC1 score and sex as fixed effects (*a priori* interaction) and colony as a random effect. There was no interaction between sex and PC1 score for bill depth ($F_{1,102}$ = 0.25, P = 0.617), bill width ($F_{1,102}$ = 1.10, P = 0.297) and head + bill length ($F_{1,102}$ = 0.07, P = 0.796) There was an interaction between sex and PC1 score for bill length ($F_{1,102}$ = 6.54, P = 0.012), with bill length increasing with PC1 score more rapidly in females than in males. Overall, these results suggest that bill allometry follows a general increase in body size when accounting for sex. The result for bill length is more difficult to interpret: sexual selection would be expected to cause bill length to increase more rapidly in males than in females and not the opposite, as here. Determining whether this particular result is an artifact of sampling will necessitate further research.

(b) Dietary study: results from stomach content and isotope analyses

Prey species composition was determined by analysis of stomach contents. It was not possible to collect stomach contents from the Mayès colony. Food samples from three previous campaigns (1997–1998, 1999–2000, 2003–2004) were thus pooled to describe bird diet at this colony. However, sex of birds from these campaigns is unknown. In all, 77 stomach contents were collected, weighing on average 70 ± 44 g. At the laboratory, the fresh fraction of contents was sorted into different prey categories. Fish species were determined according to Gon and Heemstra (1999) and our own reference collection, using the whole fish when it was preserved or the otoliths in the absence of a whole fish. All otoliths were measured (precision ± 0.01 mm), and used to estimate standard length of individual fish following Duhamel et al. (2005) and references therein.

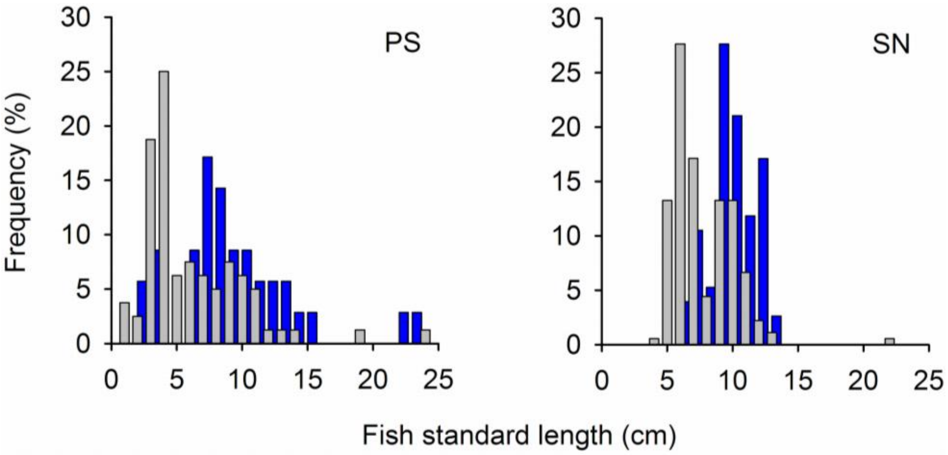

Figure B. Distribution of standard length (mouth to start of tail) of fish (all species mixed) caught by Kerguelen Shags at the Pointe Suzanne (PS) and Sourcils Noirs (SN) colonies. Females are in grey, males in blue.

Table A. Occurrence and proportion of the different prey species in Kerguelen Shags at the three study colonies. A total of 849 fish items were identified, of which 95% were benthic fish species.

		Mayès		Pointe Suzanne		Sourcils Noirs	
		Occ. (%)	Num. (%)	Occ. (%)	Num. (%)	Occ. (%)	Num. (%)
Prey type	Species	(*N* = 33)	(*n* = 376)	(*N* = 18)	(*n* = 265)	(*N* = 26)	(*n* = 310)
Fish	*Lepidonotothen mizops*	54.5	32.7	22.2	7.2	92.3	72.6
	Notothenia cyanobrancha	72.7	39.1	94.4	74.0	19.2	3.5
	Harpagifer sp.	42.4	6.1	38.9	7.2	46.1	10.0
	Gobionotothen acuta	21.2	3.7	16.7	2.6	34.6	5.8
	Channichthys rhinoceratus	12.1	1.1	–	–	3.8	0.3
	Muraenolepis marmoratus	–	–	11.1	0.7	11.5	1.0
	Paranotothenia magellanica	–	–	16.7	1.1	–	–
	Notothenia rossii	–	–	5.5	0.4	–	–
	Zanchlorhynchus spinifer	–	–	–	–	7.7	0.6
	Non identified	45.4	11.2	22.2	1.5	15.4	3.6
Annelids	Polynoidae sp.	27.3	5.3	16.7	4.9	7.7	2.3
Crustaceans	*Halicarcinus planatus*	9.1	0.8	5.5	0.4	–	–
Cephalopods	*Benthoctopus thielei*	–	–	–	–	3.8	0.3

Note: Occ. = occurrence in the stomach contents of any prey species relative to the total number of contents. *N* = total number of stomach contents. Num. = proportion of individuals from any prey species relative to the total number of prey items. *n* = total number of prey items.

An isotope analysis was performed on total blood and cover feather samples from Kerguelen Shags. Total blood informs on the type of prey the bird was feeding on during the period when the sample was taken, because of the short temporal turnover of the chemical composition of that tissue in shags. Using the allometric equations between body mass and carbon half-life in avian red blood cells found in Carleton and Martinez del Rio (2005), half-life of Kerguelen Shag total blood was estimated at 23–27 days. Feathers provide information on the bird's diet during the molt. Cover feathers of the Antarctic Blue-eyed Shag *Phalacrocorax bransfieldensis* are essentially replaced in March (Bernstein and Maxson 1981). Since all our cover feathers were collected before then and were mature and slightly worn, they represent the bird's diet during the preceding molt. Because the molt occurs just after breeding, feathers provided information on the diet immediately after the preceding breeding season, almost one year before the study. For a same bird, isotope signatures of feathers are always greater than isotope signatures of total blood, both in δ^{13}C and δ^{15}N (Quillfeldt et al. 2008). The higher the position of a prey in the food web, the more its tissues are enriched in ^{15}N (Hobson and Welch 1992). Coastal and benthic waters are more enriched in ^{13}C than offshore and pelagic waters (Cherel and Hobson 2007).

The samples for each colony and each sex were: Mayès (f [11, 11], m [8, 8]), Pointe Suzanne (f [12, 12], m [16, 18]) and Sourcils Noirs (f [14, 15], m [10, 11]) (colony (sex [number of blood samples, number of feather samples])). Samples, which were frozen in the field, were later dried for analysis. Lipids were cleaned from cover feathers using a chloroform-methanol bath. Samples were pulverized and relative abundance of stable isotopes of carbon and nitrogen were determined by continuous-flow isotope-ratio mass spectrometry. Precision of measurement was ± 0.1‰ and ± 0.3‰ for δ^{13}C and δ^{15}N, respectively.

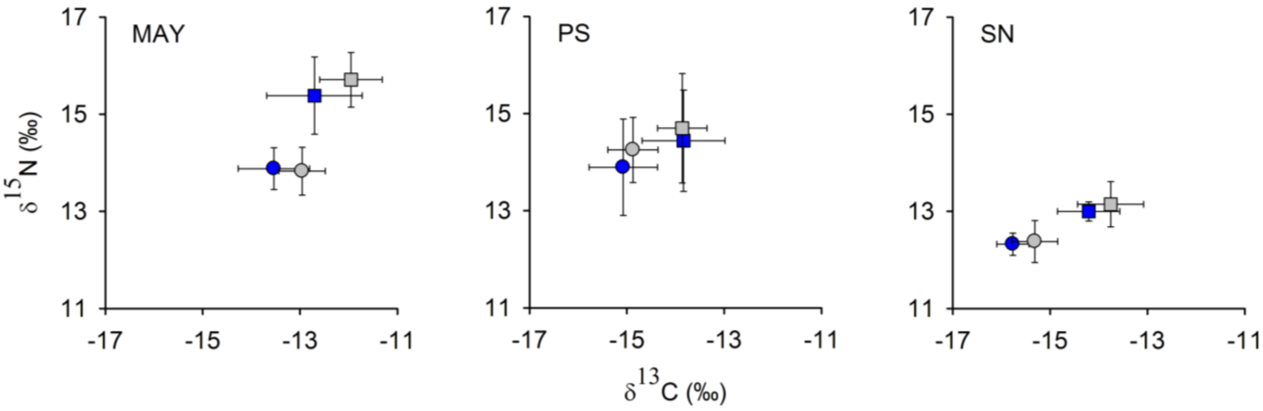

Figure C. Results from the isotope analysis performed on 71 total blood (circles) and 75 cover feather (squares) samples from Kerguelen Shags from all three study colonies. Results are presented as means ± S.E. and in the usual δ notation relative to PeeDee Belemnite and atmospheric N_2 (air) for ^{15}N/^{14}N (δ^{13}C) and ^{13}C/^{12}C (δ^{15}N) respectively. Females are in grey, males in blue.

We built a general linear model testing the effect of sex and colony (fixed effects) on the isotopic signature of Kerguelen Shag blood and feathers. There was an effect of colony on the δ^{13}C of blood ($F_{2,67}$ = 89.83, p < 0.0001) and feathers ($F_{2,67}$ = 32.17, p < 0.0001), and on the δ^{15}N of blood ($F_{2,67}$ = 50.76, p < 0.0001) and feathers ($F_{2,67}$ = 56.97, p < 0.0001). There was an effect of sex on the δ^{13}C of blood ($F_{1,67}$ = 10.50, p = 0.002) and feathers ($F_{1,67}$ = 6.22, p = 0.015), but not on the δ^{15}N of blood ($F_{1,67}$ = 0.0065, p = 0.936) or feathers ($F_{1,67}$ = 0.80, p = 0.375).

These results suggest radically different isotopic signatures of the water masses in which birds from the different colonies forage. They also suggest a spatial segregation in the feeding habitats between the sexes, males foraging further offshore compared to females. However, they do not suggest that the fish captured by males belong to a higher trophic level than those captured by females. Finally, the consistency between results obtained with the blood samples on one hand and the feather samples on the other suggests that sexual differences were conserved from one year to the next.

(c) Details on foraging behaviour: time-budget and dive parameters.

Table B. General time-budget parameters for male and female Kerguelen Shags at the three study colonies.

	Mayès		Pointe Suzanne		Sourcils Noirs	
	Females	Males	Females	Males	Females	Males
Parameter	(*N* = 5)	(*N* = 5)	(*N* = 7)	(*N* = 6)	(*N* = 11)	(*N* = 10)
Daily number of foraging trips	5.6 ± 1.6	4.1 ± 1.5	1.1 ± 0.2	1.5 ± 0.5	1.0 ± 0.1	1.2 ± 0.4
Daily time at sea (hr)	5.8 ± 1.5	7.0 ± 1.1	7.2 ± 1.2	8.2 ± 0.6	7.3 ± 0.9	6.2 ± 1.2
Daily number of dives	267 ± 92	158 ± 132	111 ± 51	45 ± 13	25 ± 5	14 ± 2
Number of chicks per nest	1.2 ± 0.4	1.2 ± 0.4	1.6 ± 0.5	1.5 ± 0.5	1.4 ± 1.0	2.0 ± 0.6
Mean chick mass (kg)	1.0 ± 0.4	1.0 ± 0.4	1.7 ± 0.0	1.7 ± 0.0	0.3 ± 0.3	0.4 ± 0.2

Note: Bird-days that did not comprise a complete recording of daily foraging activity were not included. For this reason, all data from one bird were excluded. At Sourcils Noirs, 37 % of trips were bathing trips containing no dive (first trip of the day: mean duration 7 ± 2 min); these were also excluded.

Table C. Dive parameters for male and female Kerguelen Shags at the three study colonies.

	Mayès		Pointe Suzanne		Sourcils Noirs	
	Females	Males	Females	Males	Females	Males
Parameter	(*N* = 5)	(*N* = 5)	(*N* = 8)	(*N* = 6)	(*N* = 11)	(*N* = 10)
Total number of recorded dives	2824	1210	1464	779	496	386
Maximum dive depth (m)	34	50	45	86	117	144
Maximum dive duration (s)	108	196	195	301	338	403
Maximum bottom duration (s)	72	153	164	216	219	259
Maximum post-dive interval (s)	587	595	841	1196	1354	2138
Mean dive depth (m)	6 ± 3	20 ± 8	21 ± 9	49 ± 14	91 ± 9	99 ± 12
Mean dive duration (s)	31 ± 9	92 ± 30	100 ± 32	188 ± 32	273 ± 16	296 ± 46
Mean bottom duration (s)	22 ± 6	68 ± 22	74 ± 23	124 ± 15	157 ± 14	167 ± 32
Mean post-dive interval (s)	34 ± 59	92 ± 94	99 ± 102	344 ± 186	688 ± 213	877 ± 356
Mean bottom temperature (°C)	6.5 ± 0.4	6.4 ± 0.1	5.7 ± 0.8	4.8 ± 0.3	3.9 ± 0.2	3.8 ± 0.2
Flat-bottom dives (%)	94.6	97.0	97.6	96.4	97.7	95.6

Note: Post-dive intervals that were clearly too long were likely to represent non-recovery periods at the sea surface (Tremblay et al. 2005). Therefore, a total of 83 dives were removed for calculating values of post-dive intervals. Flat-bottom dives were comprised of smooth flat-bottom dives and flat-bottom dives with wiggles.

References

Bernstein NP, Maxson SJ (1981) Notes on moult and seasonably variable characters of the Antarctic blue-eyed shag *Phalacrocorax atriceps bransfieldensis*. Notornis 28: 35–39.

Carleton SA, Martinez del Rio C (2005) The effect of cold-induced increased metabolic rate on the rate of ^{13}C and ^{15}N incorporation in house sparrows (*Passer domesticus*). Oecologia 144: 226–232.

Cherel Y, Hobson KA (2007) Geographical variation in carbon stable isotope signatures of marine predators: a tool to investigate their foraging areas in the Southern Ocean. Mar Ecol Prog Ser 329: 281–287.

Duhamel G, Gasco N, Devaine P (2005) Poissons des îles Kerguelen et Crozet. Guide régional de l'océan Austral. Paris: Muséum National d'Histoire Naturelle. 419 p.

Gon O, Heemstra PC (1999) Fishes of the southern oceans. Grahamstown: JLB Smith Institute of Ichthyology. 462 p.

Hobson KA, Welch HE (1992) Determination of trophic relationships within a high Arctic marine food web using $\delta^{13}C$ and $\delta^{15}N$ analysis. Mar Ecol Prog Ser 84: 9–18.

Quillfeldt P, Bugoni L, McGill RAR, Masello JF, Furness RW (2008) Differences in stable isotopes in blood and feathers of seabirds are consistent across species, age and latitude: implications for food web studies. Mar Biol 155: 593–598.

Smith TB (1990) Patterns of morphological and geographic variation in trophic bill morphs of the African finch *Pyrenestes*. Biol J Linn Soc 41: 381–414.

Tremblay, Y., T. R. Cook, and Y. Cherel. 2005. Time budget and diving behaviour of chick-rearing Crozet shags. Canadian Journal of Zoology 83:971–982.
