# Supplementary material for: Can Foraging Ecology Drive the Evolution of Body Size in a Diving Endotherm?
Source: PLoS One. 2013 Feb 7;8(2):e56297. doi: 10.1371/journal.pone.0056297 (PMC3567052; doi:10.1371/journal.pone.0056297)
Supplement: Appendix S2 — Literature review on Blue-eyed Shags: (a) sex-specific foraging behaviour, (b) details of studies, (c) test of Rensch’s rule, (d) effect of temperature on dive capacity, (e) effect of body mass and sex on dive capacity and (f) effect of dive depth on colony size. (PDF) [file pone.0056297.s002.pdf]

Appendix S2. Literature review on Blue-eyed Shags: (a) sex-specific foraging behaviour, (b) details of studies, (c) test of Rensch's rule, (d) effect of temperature on dive capacity, (e) effect of body mass and sex on dive capacity and (f) effect of dive depth on colony size.

(a) Sex-specific foraging behaviour

Table A. Studies on Blue-eyed Shags where sex-specific foraging behaviour was recorded.

Niche divergence	Species	Common name	Locality	Study
Prey size (♂ > ♀)	*P. bransfieldensis*	Antarctic Shag	South Shetland	Favero et al. (1998), Casaux et al. (2001)
	P. melanogenis	Crozet Shag	Crozet	Cook et al. (2007)
	P. verrucosus	Kerguelen Shag	Kerguelen	Present study
	P. purpurascens	Macquarie Shag	Macquarie	Kato et al. (1996)
Foraging time (♀ morning, ♂ afternoon)	*P. atriceps*	Imperial Shag	Falklands	Quillfeldt et al. (2011)
	P. bransfieldensis	Antarctic Shag	Antarctica / South Shetland	Bernstein and Maxson (1984) / Favero et al. (1998)
	P. georgianus	South-Georgian Shag	South Georgia	Wanless et al. (1995)
	P. melanogenis	Crozet Shag	Crozet	Cook et al. (2007)
	P. verrucosus	Kerguelen Shag	Kerguelen	Present study
	P. nivalis	Heard Shag	Heard	Green and Williams (1997)
	P. purpurascens	Macquarie Shag	Macquarie	Kato et al. (1999)
Dive depth (♂ > ♀)	*P. atriceps*	Imperial Shag	Patagonia / Falklands	Laich et al. (2011), Quintana et al. (2011) / Quillfeldt et al. (2011)
	P. georgianus	South-Georgian Shag	South Georgia	Bevan et al. (1997)
	P. melanogenis	Crozet Shag	Crozet	Cook et al. (2007)
	P. verrucosus	Kerguelen Shag	Kerguelen	Present study
	P. nivalis	Heard Shag	Heard	Green and Williams (1997)
	P. purpurascens	Macquarie Shag	Macquarie	Kato et al. (2000)

(b) Details of Blue-eyed Shag studies

Table B. Dive depth and duration for male and female Blue-eyed Shags from nine study colonies and associated colony size, air temperature and sea surface temperatures. For Punta León, only mean body mass, mean dive depth and mean duration were available in the literature. Mean body mass and SSD for Kerguelen Shags from the present study were calculated solely with individuals equipped with loggers, explaining the slight discrepancy with values in Table 1.

						Females				Males					
Locality	Colony	Size	T (°C)	SST (°C)	Breeding season	*N*	M (g)	DP (m)	DR (s)	*N*	M (g)	DP (m)	DR (s)	SSD (%)	Study
South-Georgia	Bird Island	20	1.9	2.5	1992-1993	2	2240	21	83	2	2520	23	87	12.5	Bevan et al. 1997
Macquarie	Handspike Point	364	4.8	6.6	1993-1994	12	2500	16	68	11	2910	33	115	16.5	Kato et al. 2000
Crozet	Pointe Basse	13	5.1	7.2	1998-1999	6	2100	20	87	6	2546	33	118	21.2	Cook et al. 2007
Kerguelen	Cap Cotter	200	4.6	5.6	2003-2004	5	2230	40	146	7	2643	59	196	18.5	Cook et al. 2008
Kerguelen	Mayès (North)	30	4.6	5.9	2003-2004	8	1724	16	77	6	1952	17	87	13.2	Lescroël 2005
Kerguelen	Mayès (South)	20	4.6	6.3	2005-2006	5	1547	6	32	5	1873	20	92	21.1	Present study
Kerguelen	Pointe Suzanne	450	4.6	5	2005-2006	8	2026	21	101	6	2590	49	188	27.9	Present study
Kerguelen	Sourcils Noirs	800	4.6	4	2005-2006	11	2759	91	273	10	3167	99	296	14.8	Present study
Patagonia	Punta León	3000	13.5	16	2005-2007	11	1955	29	125	9	2306	42	164	18.0	Laich et al. 2011

Note: M = mean body mass. DP = mean of mean individual dive depths. DR = mean of mean individual dive durations. T = mean annual air temperature. SST = average sea surface temperature nearby the colony during the breeding season. T was estimated using data available from World Temperature/Precipitation Data (http://bonnet19.cs.qc.edu:7778/pls/rschdata/rd_start.main), except for Macquarie (Selkirk et al. 1990), Crozet (Frénot et al. 2001) and Kerguelen (Frénot et al. 2001). SST was estimated using mean surface values recorded by the temperature sensor of loggers deployed on the study birds, except for South-Georgia (Vanyushin 2002), Macquarie (Selkirk et al. 1990) and Patagonia (Rivas 2010).

(c) Test of Rensch's rule

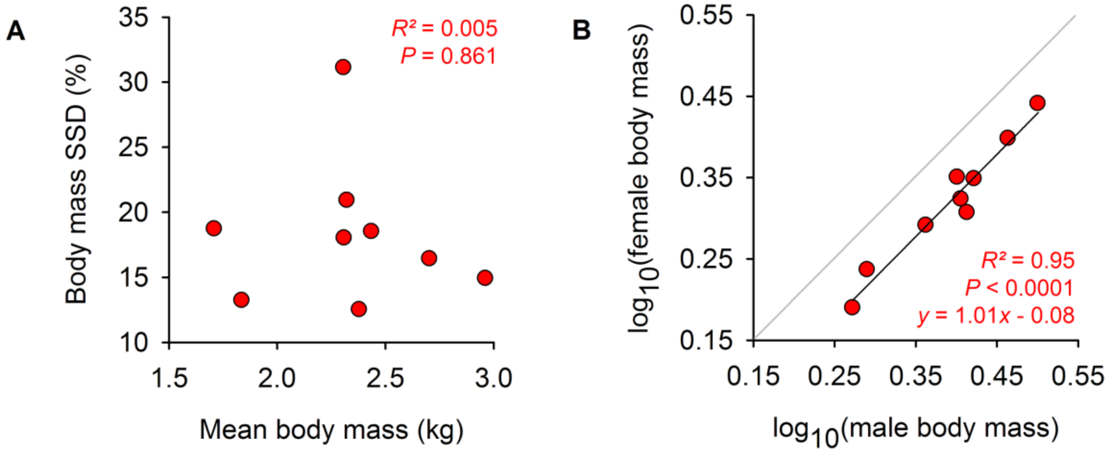

Figure A. Test of Rensch's rule across colonies of Blue-eyed Shags. (A) Effect of mean body mass on SSD (the mean body mass at any colony is the mean between average female and average male body mass). (B) Standard test of Rensch's rule using the allometric plot of $\log_{10}$(female body mass) on $\log_{10}$(male body mass). The allometry is inconsistent with Rensch's rule because the slope of the regression is > 1 (Abouheif and Fairbairn 1997). For comparison, the line of isometry is drawn ($y = x$, grey line). Rensch's rule (Rensch 1960) stipulates that SSD increases with body size under the influence of sexual selection.

(d) Effect of temperature on body size

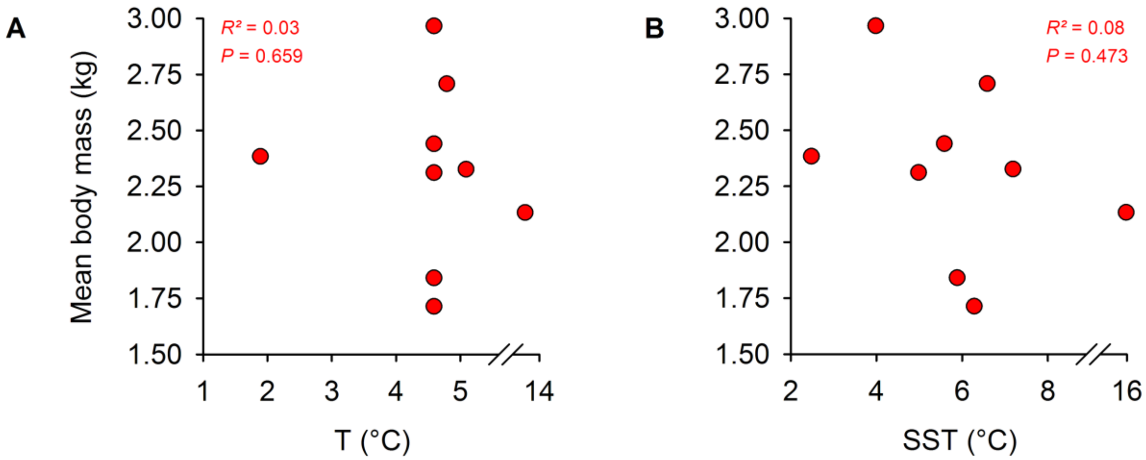

Figure B. Effect of (A) mean annual air temperature (T) and (B) sea surface temperature nearby the colony during breeding season (SST) on body mass across colonies of Blue-eyed Shags. Mean body mass for each colony was calculated as the average between female mean body mass and male mean body mass.

(e) Effect of body mass and sex on dive capacity

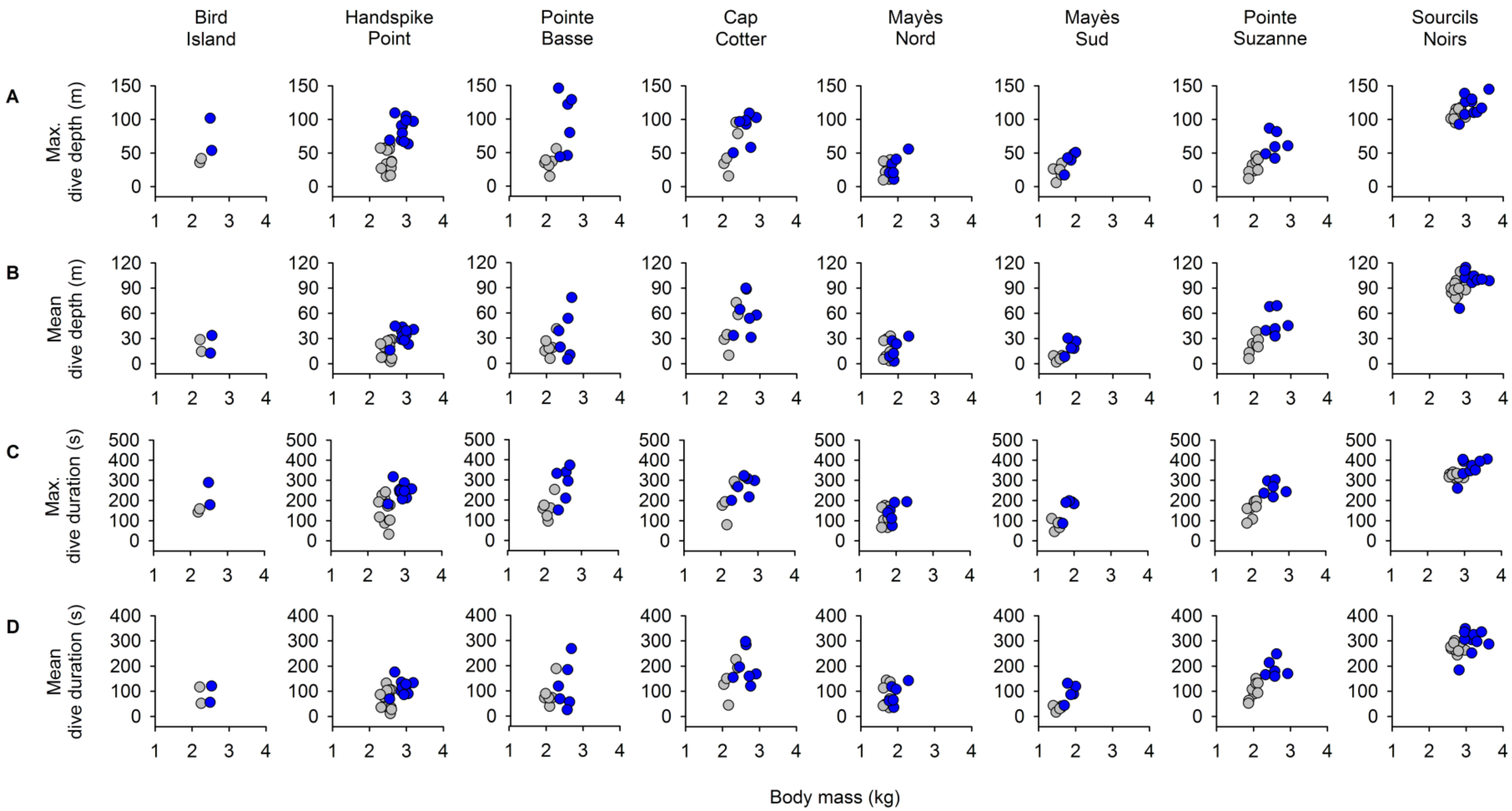

Figure C. Effect of body mass on (A) maximum dive depth, (B) mean dive depth, (C) maximum dive duration and (D) mean dive duration in both sexes for Blue-eyed Shags from eight study colonies. Females are in grey, males in blue.

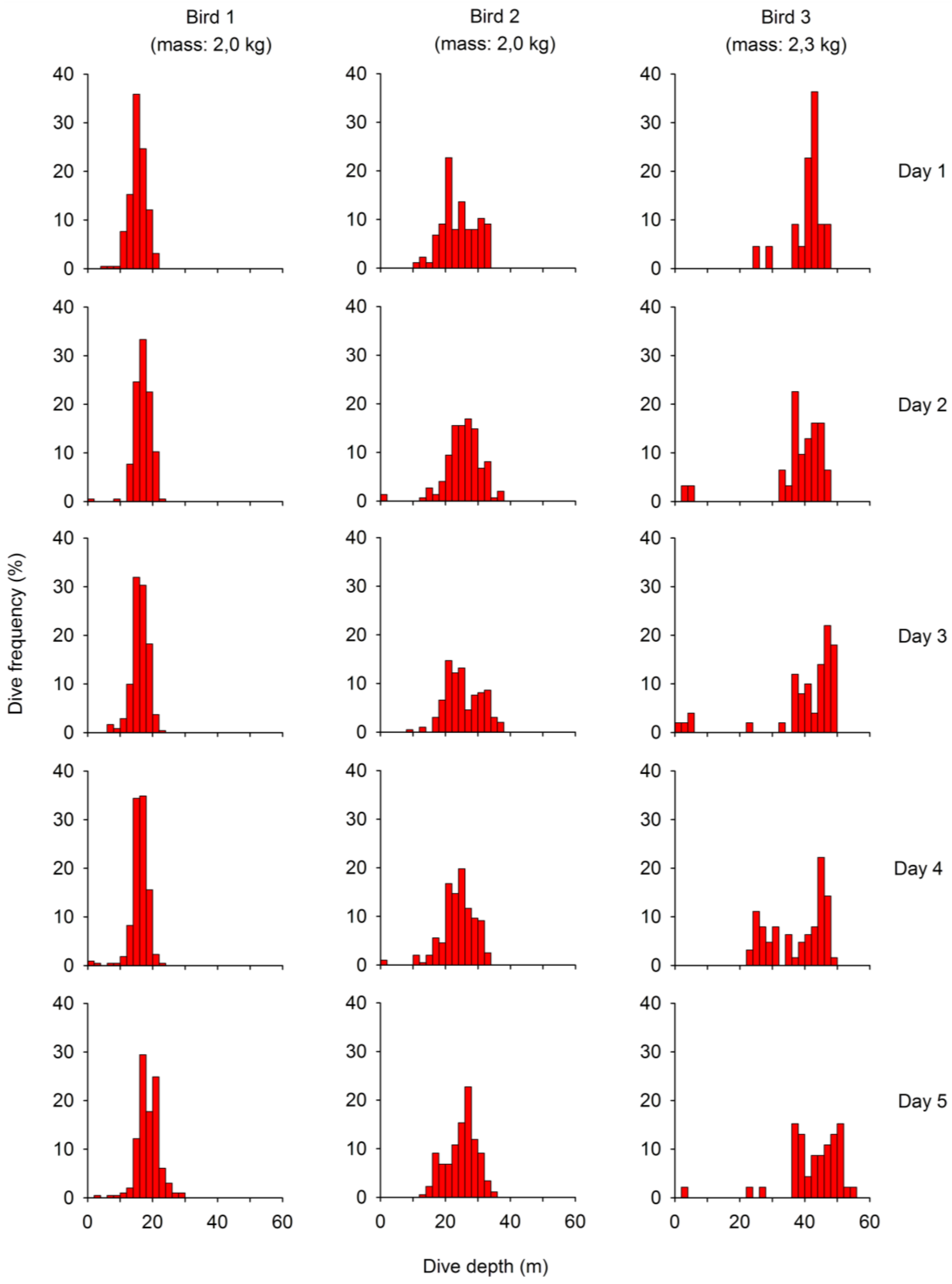

Figure D. Distribution of dive depth in three Crozet Blue-eyed Shag individuals during five consecutive days. Data from Cook et al. (2006).

(f) Effect of dive depth on colony size

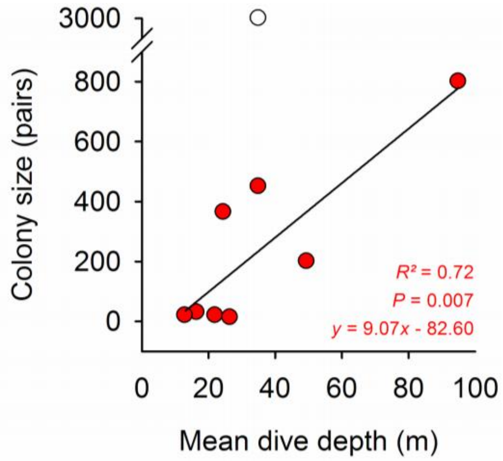

Figure E. Effect of mean dive depth on colony size across colonies of Blue-eyed Shags. The relationship was non-significant with all nine colonies (R^2 = 0.05, P = 0.546), but became significant when the Punta León colony (Table B) was removed. This was justified because birds feed on the benthic Agonidae, Clinidae, Ophidiidae and Gadidae fish at this colony, whereas they feed on the benthic notothenioid fish (Notothenidae and Harpagiferidae) at all other colonies. Furthermore, at Punta León the continental shelf is 400 km wide, whereas the insular shelf is 10–30 km wide at other colonies. The Punta León colony is shown as a white circle. As colony size is believed to be proportional to the amount of food available within foraging range, this relationship suggests that food was more abundant at greater depths.

References

Abouheif E, Fairbairn DJ (1997) A comparative analysis of allometry for sexual size dimorphism: assessing Rensch's rule. Am Nat 149: 540–562.

Bernstein NP, Maxson SJ (1984) Sexually distinct daily activity patterns of blue-eyed shags in Antarctica. Condor 86: 151–156.

Bevan RM, Boyd IL, Butler PJ, Reid K, Woakes AJ, et al. (1997) Heart rates and abdominal temperatures of free-ranging South Georgian shags, *Phalacrocorax georgianus*. J Exp Biol 200: 661–675.

Casaux R, Favero M, Silva P, Baroni A (2001) Sex differences in diving depths and diet of Antarctic shags at the South Shetland Islands. J Field Onithol 72: 22–29.

Cook TR, Cherel Y, Tremblay Y (2006) Foraging tactics of chick-rearing Crozet shags: individuals display repetitive activity and diving patterns over time. Polar Biol 29: 562–569.

Cook TR, Cherel Y, Bost C-A, Tremblay Y (2007) Chick-rearing Crozet shags (*Phalacrocorax melanogenis*) display sex-specific foraging behaviour. Antarct Sci 19: 55–63.

Cook TR., Lescroël A, Tremblay Y, Bost C-A (2008) To breathe or not to breathe? Optimal breathing, aerobic dive limit and oxygen stores in deep diving blue-eyed shags. Anim Behav 76: 265–576.

Favero M, Casaux R, Silva P, Barrera-Oro E, Coria N (1998) The diet of the Antarctic shag during summer at Nelson Island, Antarctica. Condor 100: 112–118.

Frénot Y, Gloaguen JC, Massé L, Lebouvrier M (2001) Human activities, ecosystem disturbances and plant invasions in subantarctic Crozet, Kerguelen and Amsterdam Islands. Biol Conserv 101: 33–50.

Green K, Williams R (1997) Biology of the Heard Island shag *Phalacrocorax nivalis*. 3. Foraging, diet and diving behaviour. Emu 97: 76–83.

Kato A, Nishiumi I, Naito Y (1996) Sexual differences in the diet of king cormorants at Macquarie Island. Polar Biol 16: 75–77.

Kato A, Watanuki Y, Shaughnessy P, Le Maho Y, Naito Y (1999) Intersexual differences in the diving behaviour of foraging subantarctic cormorant (*Phalacrocorax albiventer*) and Japanese cormorant (*P. filamentosus*). C R Acad Sci Paris 322: 557–562.

Kato A, Watanuki Y, Nishiumi I, Kuroki M, Shaughnessy P, et al. (2000) Variation in foraging and parental behavior of king cormorants. Auk 117: 718–730.

Laich AG, Quintana F, Shepard ELC, Wilson RP (2011) Intersexual differences in the diving behavior of Imperial Cormorants. J Ornithol 153: 139–147.

Lescroël A (2005) Foraging strategies of marine diving top-predators: comparative approach and evolutionary implications. PhD Thesis. Strasbourg: Université Louis Pasteur. 272 p.

Quillfeldt P, Schroff S, Van Noordwijk HJ, Michalik A, Ludynia K, et al. (2011) Flexible foraging behavior of a sexually dimorphic seabird: large males do not always dive deep. Mar Ecol Prog Ser 428: 271–287.

Quintana F, Wilson R, Dell'Arciprete P, Shepard E, Laich AG (2011) Women from Venus, men from Mars: inter-sex foraging differences in the imperial cormorant *Phalacrocorax atriceps*, a colonial seabird. Oikos 120: 350–358.

Rensch B (1960) Evolution above the species level. New York: Columbia University Press. 422 p.

Rivas AL (2010) Spatial and temporal variability of satellite-derived sea surface temperature in the southwestern Atlantic Ocean. Cont Shelf Res 30: 752–760.

Selkirk PM, Seppelt RD, Selkirk DR (1990) Subantarctic Macquarie Island: environment and biology. Cambridge: Cambridge University Press. 299 p.

Vanyushin GP (2002) Sea-temperature and krill catches around South Georgia in December–February 1989–1991 and 1999–2001. CCAMLR Sci 9: 165–172.

Wanless S, Harris MP, Morris JA (1995) Factors affecting daily activity budgets of South Georgian shags during chick rearing at Bird Island, South Georgia. Condor 97: 550–558.
